# Multidisciplinary approach to target volume delineation in locally recurrent rectal cancer: An explorative study

**DOI:** 10.1016/j.ctro.2025.100948

**Published:** 2025-04-01

**Authors:** F. Piqeur, D.S.C. van Gruijthuijsen, J. Nederend, H. Ceha, T. Stam, M. Dieters, P. Meijnen, M. Bakker-van der Jagt, M. Intven, A.E. Verrijssen, J.S. Cnossen, M. Berbee, M. den Hartogh, E.J. Bantema-Joppe, M. De Kroon, G. Paardekooper, M.P.M. Gielens, A.W. Daniels-Gooszen, M.J. Lahaye, D.M.J. Lambregts, S.A. Oei, J.B. Houwers, K. Horsthuis, C. Hurkmans, H. Rutten, J.W.A. Burger, C.A.M. Marijnen, H. Peulen

**Affiliations:** a Department of Radiation Oncology, Catharina Hospital, Michelangelolaan 2, 5623EJ Eindhoven, Netherlands (the); b Department of Radiation Oncology, The Netherlands Cancer Institute, Plesmanlaan 121, 1066 CX Amsterdam, Netherlands (the); c Department of Radiation Oncology, Leiden University Medical Centre, Albinusdreef 2, 2333ZA Leiden, Netherlands (the); d Department of Radiology, Catharina Hospital, Michelangelolaan 2, 5623 EJ Eindhoven, Netherlands (the); e Department of Radiation Oncology, Haaglanden Medical Centre, Burg. Banninglaan 1, 2262AK Leidschendam, Netherlands (the); f Department of Radiation Oncology, University Medical Centre Groningen, Hanzeplein 1, 9713GZ Groningen, Netherlands (the); g Department of Radiation Oncology, Amsterdam University Medical Centre, De Boelelaan 1118, 1081HZ Amsterdam, Netherlands (the); h Department of Radiation Oncology, University Medical Centre Utrecht, Heidelberglaan 100, 3584CX Utrecht, Netherlands (the); i Department of Radiation Oncology (Maastro), GROW School for Oncology and Reproduction, Maastricht University Medical Centre+, Doctor Tanslaan 12, 6229ET Maastricht, Netherlands (the); j Department of Radiation Oncology, Radiotherapiegroep, Wagnerlaan 47, 6815AD Arnhem, Netherlands (the); k Radiotherapeutisch Instituut Friesland, Borniastraat 36, 8934AD Leeuwarden, Netherlands (the); l Department of Radiation Oncology, Zuidwest Radiotherapeutisch Instituut, Boerhaavelaan 19, 4078AE Roosendaal, Netherlands (the); m Department of Radiation Oncology, Isala Zwolle, Dokter van Heesweg 2, 8025AB Zwolle, Netherlands (the); n Department of Radiology, The Netherlands Cancer Institute, Plesmanlaan 121, 1066 CX Amsterdam, Netherlands (the); o GROW School of Oncology and Developmental Biology, University of Maastricht, Universiteitssingel 40, 6229ER Maastricht, Netherlands (the); p Department of Radiology, Haaglanden Medical Centre, Burg. Banninglaan 1, 2262AK Leidschendam, Netherlands (the); q Department of Radiology, Maastricht University Medical Centre, P. Debeyelaan 25, 6229HX Maastricht, Netherlands (the); r Department of Radiology, Amsterdam University Medical Centre, De Boelelaan 1118, 1081HZ Amsterdam, Netherlands (the); s Department of Electrical Engineering and Department of Applied Physics, Eindhoven University of Technology, 5612AZ Eindhoven, Netherlands (the); t Department of Surgery, Catharina Hospital, Michelangelolaan 2, 5623EJ Eindhoven, Netherlands (the)

**Keywords:** Locally recurrent rectal cancer, Interobserver variation, Target volume delineation, Multidisciplinary collaboration

## Abstract

**Background and purpose:**

Interobserver variation (IOV) in locally recurrent rectal cancer (LRRC) delineations is large, possibly because of different interpretations of imaging. An explorative study was performed to investigate the benefit of additional delineations by expert radiologists.

**Materials and methods:**

14 cases of LRRC were delineated on planning CT by 8 radiologists (RADs) to construct a median and total radiology contour, followed by 12 radiation oncologists (ROs), without (GTV−) or with (GTV+) the additional contours. IOV was calculated separately for RADs, GTV− and GTV+. The following metrics were used: the Surface Dice Similarity Coefficient (SDSC), Dice similarity coefficient (DSC), and Hausdorff Distance at the 98th percentile (HD98%). The median SDSC, DSC, and HD98% of GTV− and GTV+ were compared. Sub-analyses of IOV in different recurrence types were performed.

**Results:**

Median SDSC significantly improved from GTV− to GTV+ overall, but a significant benefit could not be proven in individual cases. Additional radiological input consistently improved all parameters in 4/14 cases (29 %). Geographical miss occurred after radiological input in 7 %. Subgroup analyses show large IOV in mainly fibrotic and intraluminal recurrences. Little IOV is seen in solitary nodal recurrences.

**Conclusion:**

This study highlights target volume delineation challenges in LRRC. Overall, radiological input reduced IOV amongst ROs in target volume delineation for LRRC. Large differences do however exist amongst recurrence types. A standard terminology for LRRC and close collaboration between radiologists and radiation oncologists seems necessary to reduce IOV and improve quality of care.

## Highlights

• Interobserver variation in locally recurrent rectal cancer remains a clinical challenge.
• A small overall improvement is observed when delineating multidisciplinary.
• An improvement of IOV is seen in 29 % of cases when exposed to radiological contours.
• Geographical miss occurred after radiological input in 7 %.
• Sub-analyses indicate differences in interobserver variation between recurrence types.

## Introduction

Neoadjuvant chemoradiotherapy (nCRT) in locally recurrent rectal cancer (LRRC) is used to facilitate preoperative downstaging, to improve the chances of a complete surgical resection, which is the strongest predictor of oncological outcome in patients with LRRC [1], [2], [3], [4], [5], [6]. Although the use of nCRT is common, many questions still need answering, such as appropriate radiotherapy target volumes. Previously, we defined target volumes based on consensus [7]. It showed that multidisciplinary evaluation of LRRC was indispensable for delineation. Surgical input proved important for determining the clinical target volume (CTV), whereas radiological support proved important in defining the gross tumour volume (GTV) by providing background on the measure of uncertainty in imaging.

We further noticed a large inter-observer variation (IOV) amongst radiation oncologists (ROs) in delineations [7]. Especially in fibrotic tumours, this might be explained by the challenging differentiation between fibrosis and tumour [8], [9]. Efforts to reduce IOV such as adding diffusion-weighted imaging (DWI) to MRI, seem beneficial [10], [11], [12], [13]. However, the positive predictive value of MRI in determining involvement of organs can be low [14], [15].

Delineation inaccuracy is clinically relevant as it may correlate with clinical outcomes. Non-compliance with radiotherapy protocols in clinical trials has been associated with poorer oncological outcomes, specifically an increased local failure rate and decreased overall survival, e.g., as seen in the HeadSTART trial in head and neck cancer [16], [17]. In this trial, up to 25 % of reported protocol violations in the non-compliant group could be related to incorrect delineation. It is plausible that delineation inconsistencies in LRRC may also influence oncological outcomes.

IOV amongst ROs has been described in several tumour sites [16], [18]. High IOV means the likelihood of inaccurate delineations increases. It is therefore important to reduce IOV, thereby hopefully improving accuracy of delineations on a population level. Ideally, delineations would be correlated to pathological specimens to determine accuracy, but obtaining intact specimens and orientating the specimens can be very challenging, especially for LRRC (for example, in the case of multifocal recurrences or when re-resections are performed) [19].

Given the complexities and uncertainties of interpreting radiological images in LRRC, the input of a radiologist on what is deemed macroscopic tumour may reduce IOV. In this exploratory study, it is hypothesized that IOV among ROs will decrease if provided with extra radiological information in the form of a LRRC tumour demarcation by expert radiologists.

## Materials and methods

### Participant selection

The participants were recruited from PelvEx II trial centres. The PelvEx II trial is a randomized controlled trial investigating the benefit of induction chemotherapy prior to nCRT and surgery for non-metastatic LRRC [20]. Radiologists were all recruited from PelvEx II expert centres, as defined in the protocol, whereas ROs were recruited from all CRT centres.

### Case selection and preparation

Fourteen cases of LRRC were selected from a prospectively maintained LRRC database in the Catharina Hospital Eindhoven, according to the following criteria: (1) patients who underwent curative treatment for LRRC; (2) patients who received nCRT after 2016; (3) baseline imaging consisting of at least one MRI and (if applicable) imaging after induction chemotherapy of LRRC was available; (4) Complete DICOM data of nCRT for LRRC was available.

Clinical case summaries consisting of medical history and details of the local recurrence were provided. Original radiology reports were provided to ROs. Planning CTs without contrast, with a slice thickness of 3 mm were provided. MRIs were diagnostic in all cases, consisting of at least T2-weighted images and DWI. Rigid registration of planning CT to baseline MRI and, if applicable, MRI after induction chemotherapy and/or FDG-18 PET/CT at baseline and/or after induction chemotherapy was done by an experienced radiotherapy technologist. All available imaging was anonymized and distributed in a DICOM format. A summary of cases can be found in Table 1. No delineation guidelines were available, and no observer had insight into delineations performed by other participants.

**Table 1:** Summary of baseline characteristics.

#	Radiotherapy	# of lesions	Recurrence type
1	25 × 2	1	Lateral sidewall
2	15 × 2	2	Presacral in fibrosis
3	15 × 2	1	Anastomotic with presacral involvement
4	15 × 2	>3	Multifocal; along mesorectal TME-planes
5	25 × 2	2	Multifocal; presacral and central
6	25 × 2	2	Anastomotic with presacral involvement
7	15 × 2	1	Lateral nodal
8	15 × 2	1	Lateral sidewall
9	15 × 2	1	Anastomotic
10	15 × 2	1	Lateral nodal
11	15 × 2	1	Presacral in fibrosis
12	15 × 2	1	Presacral abscess
13	15 × 2	2	Multifocal; central and perineal
14	15 × 2	1	Anastomotic

### Delineation by radiologists

Expert radiologists in colorectal imaging delineated macroscopic tumour on the planning CT in all 14 cases. A median delineation was constructed using an in-house script developed in RayStation 10B-SP1, incorporating each voxel that was deemed tumour by >50 % of the radiologists. Additionally, a delineation showing each voxel that was deemed tumour by at least one radiologist was provided.

### Delineation by radiation oncologists

As in clinical practice, ROs were asked to delineate the GTV on planning CT. ROs delineated all 14 cases, of which seven without additional contours and seven with the previously constructed radiology delineations. The cases were alternated so that no RO received a case twice and so that the number of ROs delineating without (GTV− group) and with (GTV+ group) the radiology delineation was similar.

### Data analysis

Radiological IOV was calculated in reference to their median contour. IOV amongst ROs was also calculated in reference to the median of their respective group. To do so, GTV− and GTV+ median delineations were constructed following the same definition as the radiological median, i.e., incorporating each voxel deemed GTV by >50 % of the ROs of their respective group.

The following metrics were used to analyse IOV: Dice Similarity Coefficient (DSC), Surface Dice Similarity Coefficient (SDSC), and Hausdorff Distance at the 98th percentile (HD98) [19], [21]. As IOV can be described in several metrics that do not necessarily correlate with each other, the described metrics are often used to complement each other [21]. The DSC is defined as twice the intersection of two delineations divided by the sum and is a simple volume-based metric. The SDSC is defined as the agreement of the surface of two delineations within a certain threshold. A threshold of three millimetres was used, as introduced by Nikolov et al. [22] Outliers were evaluated using the Hausdorff distance (HD), defined as the maximum of all smallest distances from each point on the observer’s delineation to the median delineation. A lower HD (mm) indicates a lower variation. The HD at 100 % level is extremely sensitive to outlying points. To reduce the influence of outlying points, HD was calculated at the 98 % level (HD98%). A mean distance to agreement with standard deviation (SD) was calculated for each case and overall.

All analyses were performed using an in-house research tool developed in RayStation ® (Version 12A-SP1, RaySearch Laboratories AB, Sweden, Released 2022).

To facilitate interpretation of each metric, the following gross cut-off values were chosen; SDSC or DSC < 0.6 was categorized as large variation, 0.6 ≤ SDSC or DSC < 0.8 as average variation and ≥0.8 as little variation. A HD98% of <10 mm was classified as little variation, a HD98% ≥20 mm was defined as large variation, 10 mm ≤ HD98% <20 mm was categorized as average variation. The absolute differences in SDSC, DSC, and HD98% were calculated by subtracting the median GTV− from the median GTV+ values. To determine relevant absolute changes, a threshold of +/-0.1 was used for DSC and SDSC, and a threshold of +/-3 mm was used for HD98%.

Continuous data were reported as median (with interquartile range (IQR) and/or range). Group comparisons were performed using the Mann-Whitney *U* test. Statistical analyses were performed using IBM SPSS Statistics for Windows, Version 29.0.0.0, IBM Corp. Released 2023. Armonk, NY: IBM Corp. Comparative results were deemed statistically significant if α ≤ 0.05.

## Results

**Fig. 1:**
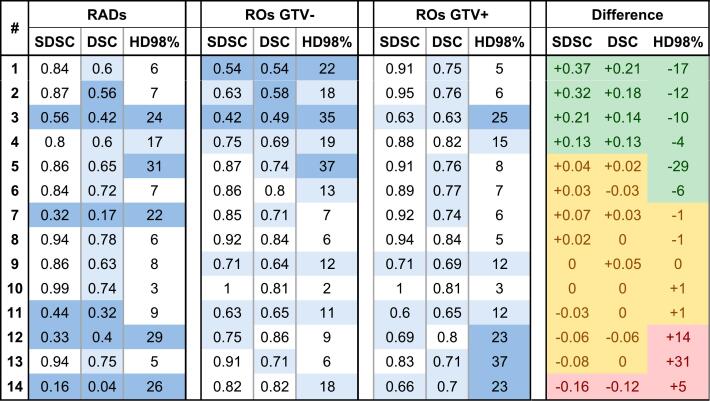
Median SDSC, DSC and HD98% for Radiologists (RAD), radiation oncologists not exposed to radiology contours (GTV−), and radiation oncologists that were exposed to the radiology contours (GTV+). Cells with large variation are marked dark blue, with average variation marked light blue, and with little variation marked white. The absolute difference between GTV+ and GTV- contours is categorized as relevant improvement (green), no relevant difference (yellow), and relevant deterioration (red).

Eight radiologists and twelve ROs participated in this study. All radiologists returned all 14 cases (112 delineations). Radiation oncologists returned 157 delineations (n = 82 GTV−, n = 75 GTV+, range 9–12 per case). Individual cases are described in the Supplementary material. The data were screened for structurally outlying observers by assessing each metric for each observer across all cases, but these were not found. Median SDSC, DSC and HD98% of each group (RADs, GTV− and GTV+), with absolute differences in each parameter (GTV+ minus GTV−), are shown in Fig. 1.

### Variation amongst radiologists

Four cases showed a large variation in all parameters amongst radiologists. Of these cases, two cases were anastomotic recurrences, and one was a recurrence located in an abscess. The large variation seen in the lateral nodal recurrence can be explained by a second lesion that was interpreted as GTV by some but considered benign by others. When disregarding this second lesion, IOV within the recurrence itself seemed little upon visual inspection.

DSC in all cases was lower amongst radiologists compared to radiation oncologists. As GTV volumes are consistently smaller amongst radiologists compared to ROs, as shown in Table S1, and DSC is heavily influenced by volume, direct comparison is not possible. Little variation is seen in seven cases based on SDSC and HD98% alone.

### Variation amongst radiation oncologists

**Fig. 2:**
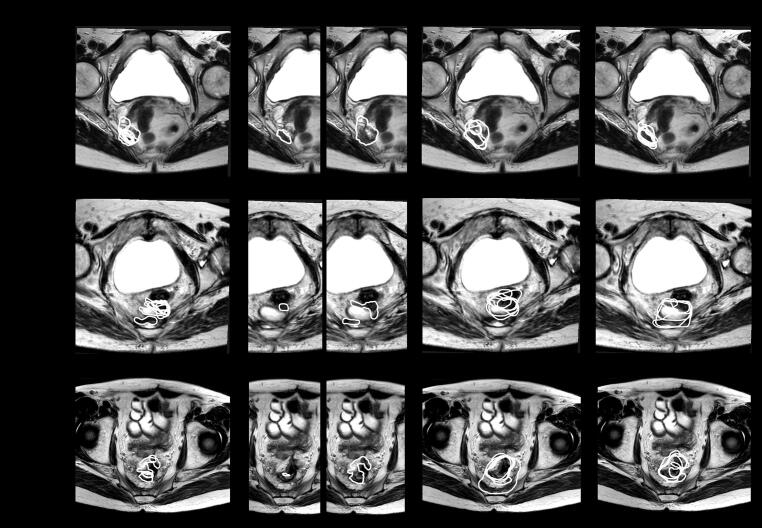
Three examples of recurrences with the constructed radiology contours and its effect on IOV. From left to right, MRI with contours by (a) radiologists, (b) median (left) and total (right) radiology contours, (c) GTV− contours and (d) GTV+ contours are shown for each case. From top to bottom, IOV decreased (case 1), remained the same (case 9) and IOV increased (case 14) from GTV− to GTV+.

In four cases, an improvement in IOV from GTV− to GTV+ that was consistent along all parameters was seen (Fig. 2), indicating a potential improvement from exposure to the radiology contours. In one case, a deterioration of all parameters was seen. Here, variation amongst radiologists was also large, possibly introducing uncertainties. In five cases, there were no relevant changes in any parameter used, indicating no benefit from exposure to radiology contours. Interestingly, three of these cases were lateral recurrences.

**Fig. 3:**
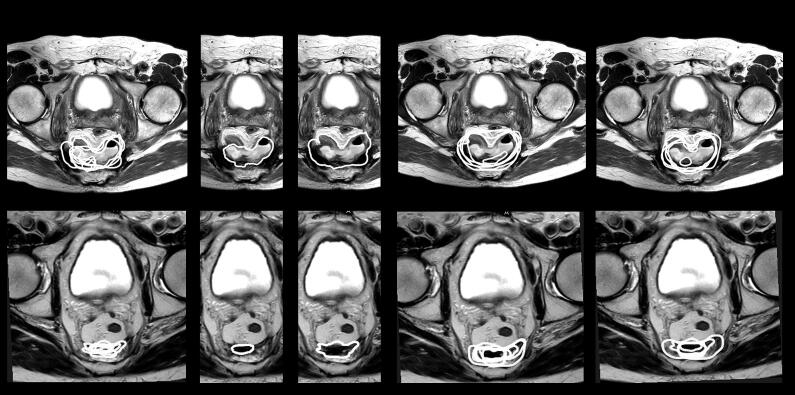
Two examples of recurrence types in which different interpretations of GTV may be explanatory for the observed IOV. From left to right, MRI is shown with (a) delineations by radiologists, (b) constructed median (left) and total (right) radiology contours, (c) delineations by GTV− group and (d) delineations by GTV+ group. The top row shows a recurrence located within a presacral abscess. The bottom row shows a presacral, fibrotic tumour. In both cases, observers either delineated the whole abscess (RADs 4/8; GTV− 5/6; GTV+ 4/5) or fibrosis (RADs 3/8; GTV− 4/6; GTV+ 3/5), or delineated tumour within the abscess (RADs 4/8; GTV− 1/6; GTV+ 1/5) or fibrosis (RADs 5/8; GTV− 2/6; GTV+ 2/5). In both cases, no difference in IOV with or without radiology contours was seen.

In the four remaining cases, differences could only be detected in the median HD98%, ranging from an improvement of 29 mm up to a deterioration of 31 mm. In one of these cases, a second perineal lesion was missed by all radiologists. All ROs (n = 5) in the GTV− group contoured both lesions. In contrast, in the GTV+ group, 4/6 ROs did not contour the perineal lesion, possibly influenced by the radiological miss, causing the median HD98% to increase steeply. Three examples of recurrences in which exposure to the radiology contours decreased IOV, increased IOV and had no influence on IOV are shown in Fig. 3.

Overall, a mean distance to agreement of 3.86 mm was seen amongst all radiation oncologists, with an overall SD of 2.27 mm, as shown in Table S2.

The observed differences were not statistically significant for any single parameter in individual cases, except a significant decrease of median HD98% in case 1 (22 mm GTV− to 5 mm GTV+, p = 0.002). When comparing all GTV− versus all GTV+ contours, only the improvement in SDSC reached statistical significance, as shown in Table 2. When excluding cases where radiologists demonstrated large variation (possibly introducing more uncertainties), overall SDSC, DSC and HD98% all significantly improved in the GTV+ group versus the GTV− group, indicating a benefit of the exposure to radiological delineations (Table S3).

**Table 2:** Overall median SDSC, DSC and HD98% (mm) of all GTV− contours and GTV+ contours.

		SDSC	IQR	DSC	IQR	HD98	IQR
All RO contours (n = 157)	GTV−	0.84	0.60–0.91	0.72	0.60–0.81	11	6–23
	GTV+	0.88	0.69–0.95	0.76	0.65–0.82	7	5–21
	*p-value*	**0.019**		**0.116**		**0.075**	

### Variation in recurrence types

Characteristics and focality of recurrences seem to influence IOV (Table 3). The largest IOV is seen in fibrotic recurrences, followed by intraluminal recurrences. The smallest IOV is seen in lateral recurrences, specifically solitary nodal recurrences. The same pattern is seen amongst radiologists (Table S4).

**Table 3:** IOV per group in different recurrence types, categorized as seen in Table 1. The largest IOV is seen in intraluminal and fibrotic recurrences. The smallest IOV is seen in lateral recurrences, specifically in solitary lymph node recurrences. Comparisons are made between median SDSC, DSC and HD98% for GTV− contours and GTV+ contours.

		GTV−		GTV+		p-value
		n=	Median	n=	Median	
**Lateral recurrence**	SDSC	23	0.85	22	0.95	0.060
*4 cases included*	DSC		0.72		0.79	0.199
	HD98		7		5	**0.024**
**Solitary lymph node recurrence**	SDSC	11	0.94	10	0.96	0.756
*2 cases included*	DSC		0.72		0.79	0.654
	HD98		6		5	0.918
**Multifocal recurrence**	SDSC	27	0.86	26	0.89	**0.040**
*5 cases included*	DSC		0.73		0.76	0.072
	HD98		13		7	0.107
**Anastomotic recurrence**	SDSC	24	0.77	21	0.77	0.741
*4 cases included*	DSC		0.72		0.75	0.750
	HD98		14		12	0.517
**Fibrotic recurrence**	SDSC	24	0.58	21	0.69	0.155
*4 cases included*	DSC		0.59		0.67	0.487
	HD98		14		12	0.495

A: Cases included in the analysis of lateral recurrences: 1, 7, 8, 10.

B: Cases included in the analysis of solitary lymph node recurrences: 7, 10.

C: Cases included in the analysis of multifocal recurrences: 2, 4, 5, 6, 13.

D: Cases included in the analysis of intraluminal recurrences: 3, 6, 9, 14.

E: Cases included in the analysis of fibrotic recurrences: 2, 3, 11, 12.

## Discussion

This study is, to our knowledge, the first to specifically target IOV in LRRC. Our results suggest that providing radiological delineations can reduce IOV, mainly when there is a large up-front IOV amongst ROs. No benefit is reported in cases where overall agreement is already high (i.e., little IOV), as seen in solitary nodal recurrences. Certain recurrence types (such as fibrotic recurrences) result in large IOV amongst radiologists and ROs. For these subtypes, a multidisciplinary approach with optimal integration of all available clinical and radiological information seems crucial in optimizing delineations.

Overall, a significant improvement in surface agreement (SDSC) is seen when comparing all GTV+ contours with GTV− contours. No significant improvement is seen when comparing volumetric agreement (DSC) or outlying boundaries (HD98%) of all GTV− contours with GTV. An effect in DSC and HD98% can be seen when excluding cases in which radiologists showed large interobserver variation. By reducing IOV, the chance of inaccurate delineations may decrease, however there is no way verify the accuracy (or quality) of the performed contours, as it not feasible to correlate the delineations to pathology. The observed reduction of SDSC may be a sign that a multidisciplinary approach to delineation can aid in target volume delineations for LRRC patients. The effect is, however, only small and varies significantly between individual cases.

In four cases an improvement was seen in all parameters. When visually inspecting these cases, we could see less tumour miss (compared to the radiology contours) (n = 2) and significantly smaller GTVs (n = 2) in the GTV+ delineations versus the GTV− delineations. In case 4, 4/5 GTV− contours contained tumour miss when comparing them to the radiology contours versus 0/4 in the GTV+ contours. In this study therefore, a reduction in IOV may also translate in a clinical benefit in 29 % of cases. This would be in line with a previous study by Dimigen et al., that reported changes in target volumes in up to 25 % of cases when a radiologist is incorporated into the radiation oncology workflow [23]. We would therefore recommend consultation with dedicated radiologists when determining target volumes for LRRC. However, we also observed geographical miss due to radiological input in one case (7 %), with potential clinical harm, therefore further optimisation of the collaboration and delineation process is needed.

A first step in optimizing the delineation process may be to develop a common language for LRRC. As shown in Fig. 3, differences in interpretation of GTV in, for example, fibrotic tumours and tumours located in an abscess may have hampered IOV results amongst both radiologists and radiation oncologists. The lack of a standard definition of GTV is not a new phenomenon and is seen in several tumour types [16]. In primary rectal cancer, Sluckin et al. detailed several differences in the anatomical interpretation of lateral lymph nodes amongst the disciplines involved, possibly complicating multidisciplinary communication [24]. Given the complexity and heterogeneity of LRRC, due to factors such as differences in recurrence locations and types, differences in previous treatment, altered anatomy after surgery, and the loss of normal anatomical boundaries, it is highly likely that interpretation and nomenclature differences are even more substantial in LRRC than in primary rectal cancer.

Ideally, the aim should be to reach consensus on the interpretation of the GTV amongst physicians, to ensure we are ‘speaking the same language’, and to adjust radiological aid and reporting accordingly. The ESGAR, ESUR and PelvEx collaborative groups are standardizing radiological reporting for patients that are planned to undergo a pelvic exenteration, such as for LRRC [25]. Given the overlap in patient groups, this standardized reporting may also be beneficial for collaboration amongst ROs and radiologists. As of now, we would advise radiologists to detail all (possibly) involved structures and indicate a measure of uncertainty. Demarcations of involved and at-risk areas within the small pelvis may prove helpful in determining tumour extent.

In addition to a standard definition of GTV, a broad consensus on target volumes should be reached. Parallel to the timeframe of this study, a multidisciplinary delineation guideline for LRRC was developed [7], [20]. Some differences in GTV interpretation were addressed, such as how to delineate fibrotic tumours, which may have reduced IOV in these cases. However, some observed differences are yet to be specified, such as whether or not to delineate the whole lumen as GTV in anastomotic recurrences. In these instances, the aid of a radiological tumour demarcation may only be useful after a common interpretation of GTV is established [18]. The current study may help in the development of the guideline by highlighting areas of disagreement.

The established guideline combined with prospective peer-review of delineations is currently being evaluated within the PelvEx II trial [20]. In primary rectal cancer, central review of delineations in the PROCARE study led to alterations in up to 74 % of cases [26]. Moreover, the PROCARE study enabled identification of subregions with the least contouring agreement. Subsequent refinement of the consensus guidelines led to higher uniformity in delineations and led to a decrease in the number of alterations to 53 % [27]. Quality assurance in the PelvEx II trial will hopefully lead to similar results, by facilitating guideline development and improving uniformity of CRT treatment. Preliminary data from the PelvEx II study have already shown that target volumes are altered in up to 50 % of cases after peer-review for LRRC [28].

Lastly, we would propose working towards agreement on which imaging to use and how to ensure proper visibility and comparability. MRI is the gold-standard modality for locoregional evaluation of LRRC, due to its superior soft tissue visualisation [8], [9], [29], [30]. However, contouring and treatment planning for LRRC is often CT-based. Matching diagnostic MRI to CT is an easy way to facilitate contouring but may lead to setup errors, even more so with angulated images, also complicating comparability. Incorporating an MRI in radiotherapy position into the standard radiotherapy workflow for LRRC patients is a robust way to aid clinicians in delineation.

Performed subgroup analyses show interesting differences in IOV amongst recurrence types. In solitary nodal recurrences, very little IOV was seen, explaining why no further improvement was seen when comparing GTV− to GTV+, whereas in recurrences located in fibrosis, much larger IOV was seen. Although the groups are small, limiting conclusions, there is a suggestion of an improved IOV from GTV− to GTV+ due to radiological aid, mainly in fibrotic recurrences (SDSC 0.58 to 0.69). Multidisciplinary delineations may therefore still be beneficial, but with more clinical and numerical effect in certain recurrence types than others.

There are several limitations to the current study. As not all ROs returned all cases, the number of contours per group varied, potentially influencing results both for better and for worse. The total number of participants was large, however the number of ROs per group was small, limiting comparisons. Median and total radiology contours were constructed from 8 delineations performed by expert radiologists. Nevertheless, there is no way to guarantee that these delineations are truer than delineations performed by ROs. Moreover, large IOV amongst radiologists is also reported, and this approach can introduce errors such as tumour miss, as reported. Radiologists further mentioned they were hindered by the lack of repeated imaging, complicating recognition. Prior imaging was often unavailable, due to the patient selection from our tertiary referral centre, where patients are discussed after LRRC diagnosis rather than during follow-up, and previous imaging is therefore often not retrieved unless there is clinical doubt.

At the time of the initiation of this study, no formal delineation guidelines or reporting guidelines for LRRC were available. All observers were therefore instructed to delineate primarily on planning CT, as in clinical practice, but without a guideline detailing GTV definition. As discussed earlier, this can lessen the effect of radiological support, as differing interpretations of GTV seemed more influential than spatial disagreement in several cases. Lastly, IOV is described in metrics, each with their own strengths and weaknesses. By combining three metrics and focussing on differences that were consistent along all three metrics, we have tried to overcome these issues.

## Conclusion

This study highlights target volume delineation challenges in LRRC. Overall, radiological input reduced interobserver variation amongst radiation oncologists in target volume delineation for LRRC. Large differences do however exist amongst recurrence types. A standard terminology for LRRC and close collaboration between radiologists and radiation oncologists seems necessary to reduce IOV and improve quality of care.

## CRediT authorship contribution statement

**F. Piqeur:** Conceptualization, Data curation, Formal analysis, Investigation, Methodology, Visualization, Writing – original draft. **D.S.C. van Gruijthuijsen:** Formal analysis, Methodology, Software, Writing – review & editing. **J. Nederend:** Conceptualization, Investigation, Writing – review & editing. **H. Ceha:** Investigation, Writing – review & editing. **T. Stam:** Investigation, Writing – review & editing. **M. Dieters:** Investigation, Writing – review & editing. **P. Meijnen:** Investigation, Writing – review & editing. **M. Bakker-van der Jagt:** Investigation, Writing – review & editing. **M. Intven:** Investigation, Writing – review & editing. **A.E. Verrijssen:** Investigation, Writing – review & editing. **J.S. Cnossen:** Investigation, Writing – review & editing. **M. Berbee:** Investigation, Writing – review & editing. **M. den Hartogh:** Investigation, Writing – review & editing. **E.J. Bantema-Joppe:** Investigation, Writing – review & editing. **M. De Kroon:** Investigation, Writing – review & editing. **G. Paardekooper:** Investigation, Writing – review & editing. **M.P.M. Gielens:** Investigation, Writing – review & editing. **A.W. Daniels-Gooszen:** Investigation, Writing – review & editing. **M.J. Lahaye:** Investigation, Writing – review & editing. **D.M.J. Lambregts:** Investigation, Writing – review & editing. **S.A. Oei:** Investigation, Writing – review & editing. **J.B. Houwers:** Investigation, Writing – review & editing. **K. Horsthuis:** Investigation, Writing – review & editing. **C. Hurkmans:** Methodology, Software, Writing – review & editing. **H. Rutten:** Conceptualization, Supervision, Writing – review & editing. **J.W.A. Burger:** Project administration, Resources, Supervision, Writing – review & editing. **C.A.M. Marijnen:** Conceptualization, Supervision, Writing – review & editing. **H. Peulen:** Conceptualization, Project administration, Supervision, Writing – review & editing.

## Declaration of competing interest

The authors declare that they have no known competing financial interests or personal relationships that could have appeared to influence the work reported in this paper.
