# Supplementary material for: Multidisciplinary approach to target volume delineation in locally recurrent rectal cancer: An explorative study
Source: Clin Transl Radiat Oncol. 2025 Apr 1;53:100948. doi: 10.1016/j.ctro.2025.100948 (PMC12017975; doi:10.1016/j.ctro.2025.100948)
Supplement: Supplementary Data 1 [file mmc1.docx]

**Supplementary material**

[Supplementary tables and figures 2](#_Toc152929263)

[Case information 3](#_Toc152929264)

[Case 1 3](#_Toc152929265)

[Case 2 5](#_Toc152929266)

[Case 3 7](#_Toc152929267)

[Case 4 9](#_Toc152929268)

[Case 5 11](#_Toc152929269)

[Case 6 13](#_Toc152929270)

[Case 7 15](#_Toc152929271)

[Case 8 17](#_Toc152929272)

[Case 9 19](#_Toc152929273)

[Case 10 21](#_Toc152929274)

[Case 11 23](#_Toc152929275)

[Case 12 26](#_Toc152929276)

[Case 13 28](#_Toc152929277)

[Case 14 30](#_Toc152929278)

**Supplementary tables and figures**

**Table S1.** Median volume (with IQR) amongst radiologists and radiation oncologists per case. For radiation oncologists, median volume of both GTV- and GTV+ group contours is also provided.

| **#** | **Radiologists** | | **Radiation Oncologists** | | | |
| --- | --- | --- | --- | --- | --- | --- |
|  | **Median** | **IQR** | **Median** | **IQR** | **Median GTV-** | **Median GTV+** |
| 1 | 3.55 | 2,17-4,45 | 8.80 | 5,79-24,68 | 21.85 | 5.92 |
| 2 | 2.26 | 1,23-3,37 | 5.56 | 4,50-16,06 | 12.93 | 4.50 |
| 3 | 13.93 | 3,72-22,69 | 42.61 | 17,49-91,79 | 59.53 | 27.35 |
| 4 | 34.25 | 21,02-48,73 | 90.67 | 58,51-105,21 | 83.00 | 91.26 |
| 5 | 5.63 | 3,92-6,38 | 10.82 | 9,96-15,06 | 10.73 | 11.57 |
| 6 | 17.09 | 12,15-21,58 | 27.77 | 19,98-32,40 | 29.82 | 19.98 |
| 7 | 1.67 | 1,36-2,44 | 4.48 | 2,54-6,14 | 4.55 | 4.48 |
| 8 | 6.03 | 2,05-7,00 | 9.24 | 7,39-10,90 | 9.11 | 10.20 |
| 9 | 4.18 | 3,45-4,69 | 11.96 | 9,40-21,88 | 11.02 | 19.34 |
| 10 | 0.99 | 0,87-1,26 | 1.49 | 1,27-1,85 | 1.37 | 1.50 |
| 11 | 1.94 | 0,54-6,78 | 15.00 | 6,85-18,04 | 15.64 | 13.58 |
| 12 | 53.11 | 5,30-100,85 | 138.42 | 107,98-204,38 | 180.26 | 115.33 |
| 13 | 2.70 | 2,15-3,30 | 6.22 | 4,35-8,94 | 8.81 | 5.80 |
| 14 | 2.11 | 0,99-4,70 | 34.49 | 12,59-44,26 | 34.49 | 32.18 |

**Table S2**: Mean distance to agreement (MDA) and standard deviation (SD) per case of all radiation oncologists in reference to the median GTV of all radiation oncologists. An overall mean and standard deviation of all MDAs is also given.

| **#** | **RT** | |
| --- | --- | --- |
|  | **MDA (mm)** | **SD** |
| 1 | 5.39 | 5.65 |
| 2 | 4.45 | 4.66 |
| 3 | 8.97 | 5.64 |
| 4 | 2.97 | 0.72 |
| 5 | 2.14 | 1.01 |
| 6 | 1.87 | 0.73 |
| 7 | 2.56 | 2.71 |
| 8 | 1.49 | 0.53 |
| 9 | 3.46 | 1.39 |
| 10 | 0.88 | 0.21 |
| 11 | 3.30 | 1.72 |
| 12 | 7.46 | 8.61 |
| 13 | 3.53 | 2.69 |
| 14 | 4.83 | 4.35 |
| Overall | **3.86** | **2.27** |

**Table S3**: Overall median SDSC, DSC and HD98% (mm) of GTV- contours and GTV+ contours, when excluding cases with large IOV among radiologists (case 3, 7, 12, 14).

|  |  | **SDSC** | **IQR** | **DSC** | **IQR** | **HD98** | **IQR** |
| --- | --- | --- | --- | --- | --- | --- | --- |
| **Excluding cases 3, 7, 12, 14 (n=109)** | GTV- | 0.84 | 0.65-0.92 | 0.73 | 0.62-0.80 | 11 | 6-17 |
|  | GTV+ | 0.89 | 0.80-0.97 | 0.77 | 0.68-0.82 | 6 | 4-12 |
|  | *p-value* | **0.008** |  | **0.026** |  | **0.016** |  |
|  |  |  |  |  |  |  |  |

**Table S4**. IOV amongst radiologists in different recurrence types, categorized as seen in table 1. Highest IOV is seen in intraluminal and fibrotic recurrences. Lowest IOV is seen in lateral recurrences, but more specifically in solitary lymph node recurrences.

| **IOV amongst radiologists** | | **n=** | **Median** |
| --- | --- | --- | --- |
| **Lateral recurrence** | SDSC | 32 | 0.90 |
| *4 cases included* | DSC |  | 0.67 |
|  | HD98 |  | 6.0 |
| **Solitary lymph node recurrence** | SDSC | 16 | 0.82 |
| *2 cases included* | DSC |  | 0.48 |
|  | HD98 |  | 5.7 |
| **Multifocal recurrence** | SDSC | 40 | 0.86 |
| *5 cases included* | DSC |  | 0.66 |
|  | HD98 |  | 10.0 |
| **Intraluminal recurrence** | SDSC | 32 | 0.62 |
| *4 cases included* | DSC |  | 0.47 |
|  | HD98 |  | 14.3 |
| **Fibrotic recurrence** | SDSC | 32 | 0.64 |
| *4 cases included* | DSC |  | 0.51 |
|  | HD98 |  | 18.90 |

**Case information**

A summary of case information and available radiology reports is provided per case, followed by radiological imaging. Contours by radiologists, with the constructed gold standard (i.e. median GTV of radiologists) and total GTV (i.e., all voxels incorporated into the GTV by at least one radiologist), and contours by radiation oncologists (GTV- and GTV+) are shown. Volume, SDSC, DSC and HD98% per case are shown.

**Case 1**

71-year old female, presenting with a local recurrence located on the right side of the pelvic floor, 2 years after a T2N0 rectal cancer, for which an APR was performed. Patient has received three cycles of CAPOX and has now been referred for neoadjuvant CRT. Patient is planned for surgical resection of the lateral side wall with IORT.

Imaging:

- *Baseline MRI:* Suspicion of a local recurrence on the right pelvic side wall.
- *Baseline PET-CT:* Suspicion of a local recurrence as described on MRI. No pathological lymph nodes or distant metastases observed.
- *PET-CT after induction chemotherapy:* Partial metabolic response with a decrease in SUV but a slight increase in size.


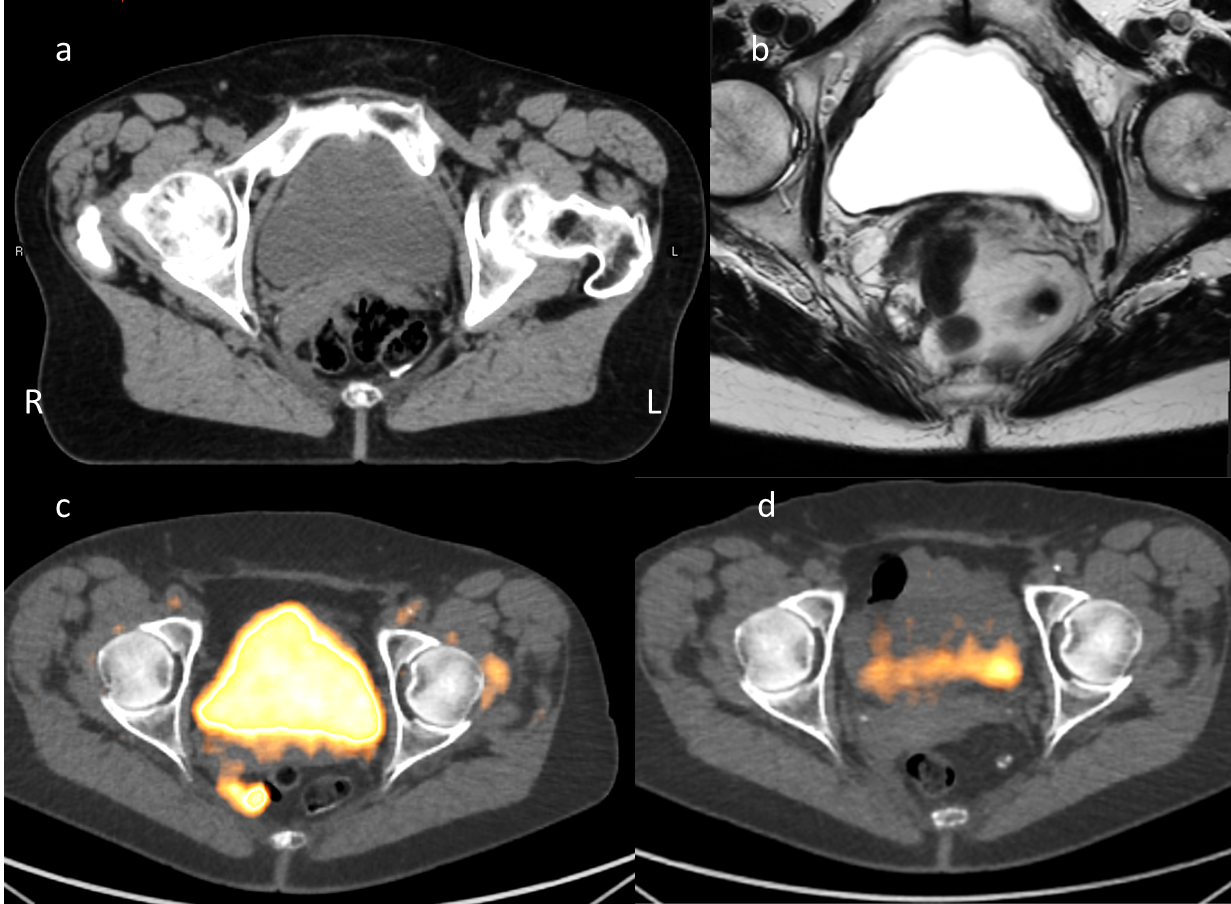


Figure showing (a) Planning CT, (b) MRI at baseline (c) PET-CT prior to induction chemotherapy (d) PET-CT following induction chemotherapy


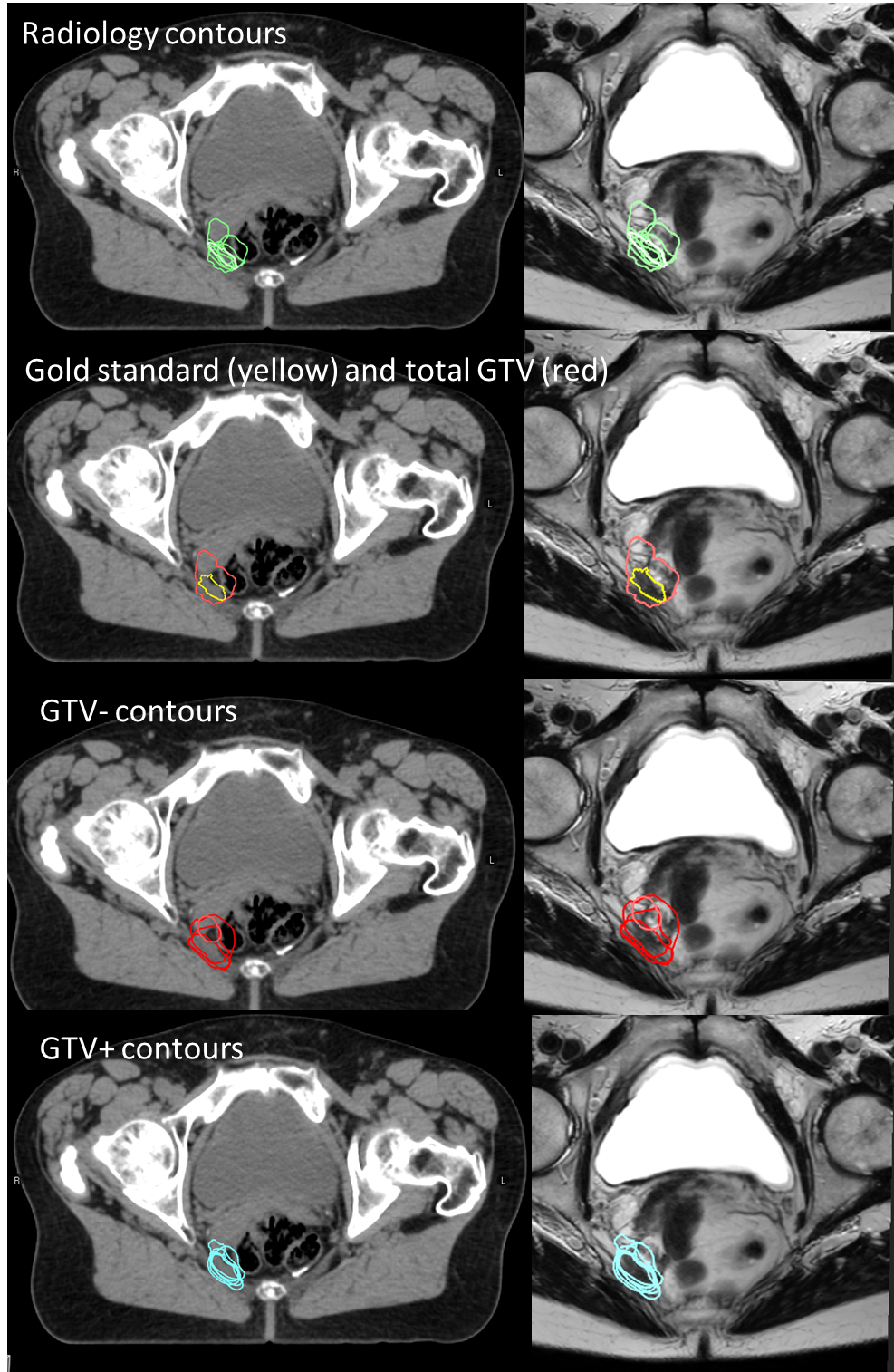


| **Case 1** | **RAD (n=8)** | | **GTV- (n=6)** | | **GTV+ (n=6)** | |
| --- | --- | --- | --- | --- | --- | --- |
|  | **Median** | **Range** | **Median** | **Range** | **Median** | **Range** |
| Volume (cc) | 3.5 | 1.5-5.1 | 21.9 | 9.6-42.6 | 5.9 | 3.3-8.0 |
| SDSC (0-1) | 0.84 | 0.25-0.96 | 0.54 | 0.36-0.85 | 0.91 | 0.80-0.97 |
| DSC (0-1) | 0.60 | 0.08-0.72 | 0.54 | 0.30-0.79 | 0.75 | 0.61-0.84 |
| HD98% (mm) | 6.3 | 3.8-22.3 | 21.8 | 7.9-38.5 | 4.6 | 3.4-6.1 |

**Case 2**

71-year old male, with a history of a T3N2M1 rectal cancer (with a solitary liver metastasis). Patient was previously treated with 5x5Gy radiotherapy and 8 cycles of CAPOX, followed by ablation of the liver metastasis and an APR (pT3N2, R0). Patient was referred with a multifocal recurrence and has received 4 cycles of FOLFIRI. Patient is planned for surgical resection and intra-operative radiotherapy after chemo reirradiation.

Imaging:

- *Baseline MRI:* Two locations of local recurrence: nodal recurrence ventral from L5-S1, local recurrence ventral from S4-S5.
- *Baseline PET-CT:* Suspicion of a lymph node metastasis in the trajectory of the inferior mesenteric vessels. Pre-coccygeal FDG-avid mass seen, possibly showing a presacral recurrence.
- *PET-CT after induction chemotherapy:* Good metabolic response of the mesenteric lymph nodes and presacral lesion. No new lesions seen.


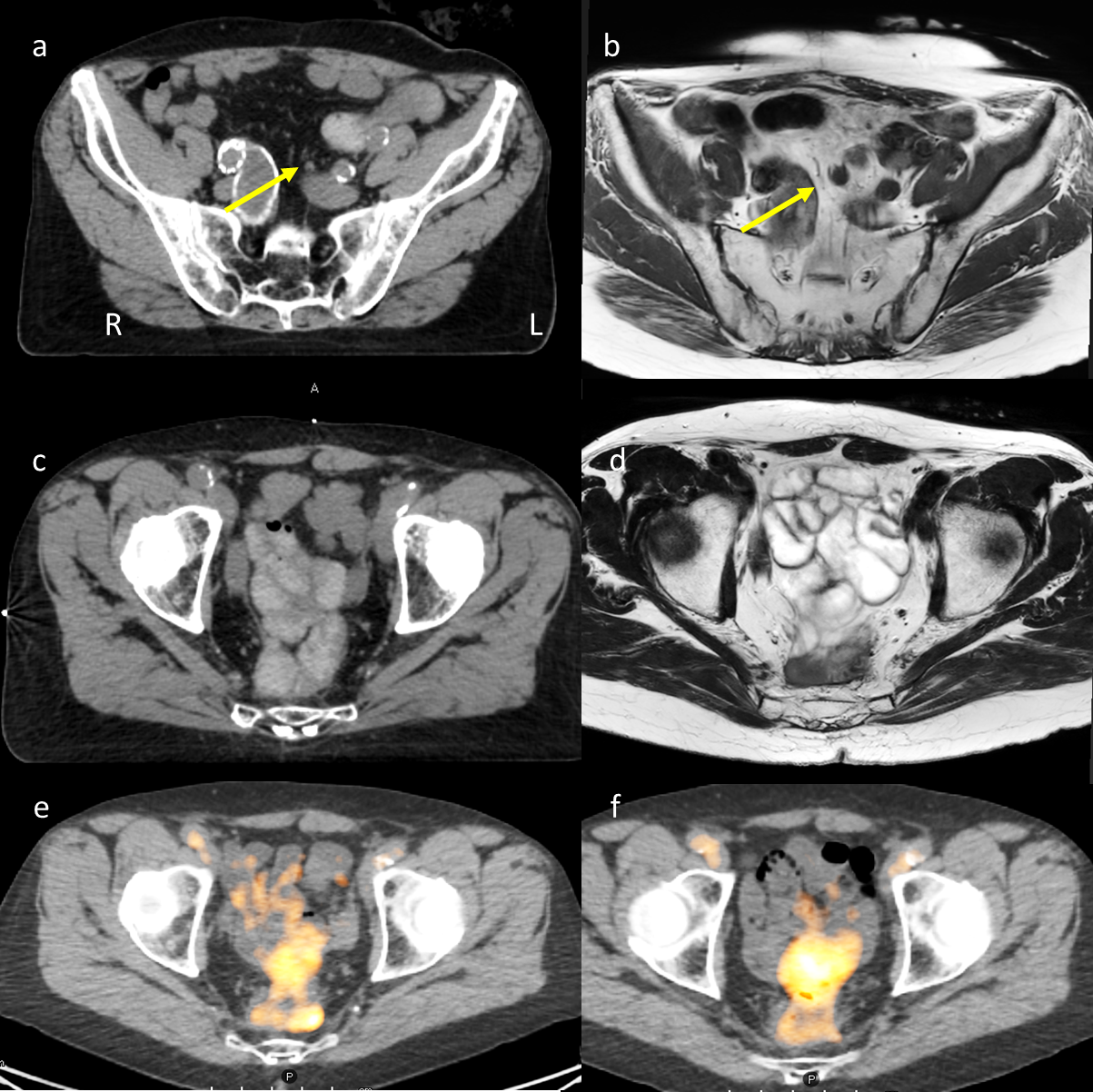


Figure showing the lymph node recurrence on planning CT (a) and T1W MRI (b), and showing the presacral recurrence on planning CT (c), MRI (d), PET-CT before induction chemotherapy (e) and after induction chemotherapy (f).


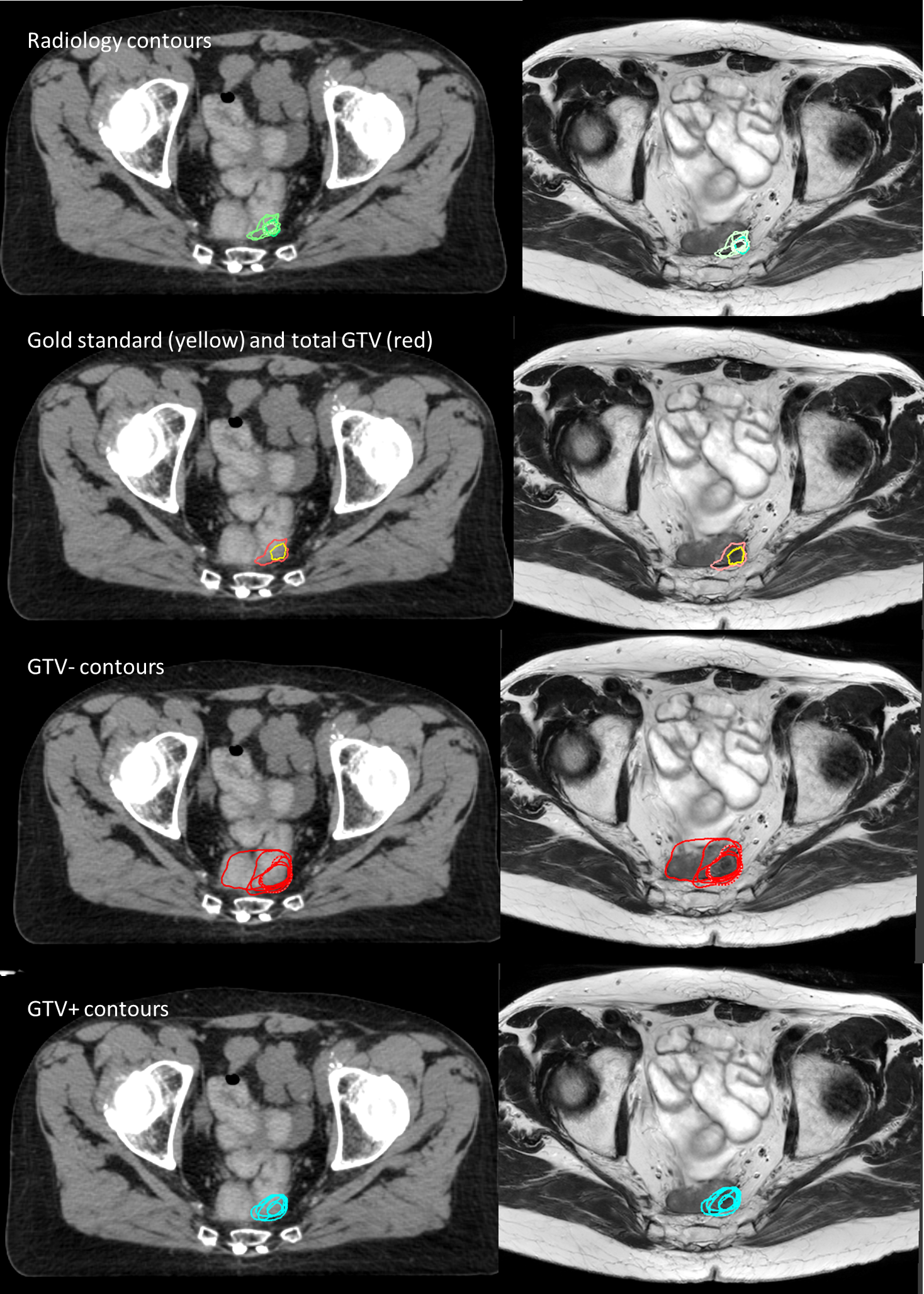


| **Case 2** | **RAD (n=8)** | | **GTV- (n=6)** | | **GTV+ (n=5)** | |
| --- | --- | --- | --- | --- | --- | --- |
|  | **Median** | **Range** | **Median** | **Range** | **Median** | **Range** |
| Volume (cc) | 2.3 | 0.8-5.3 | 12.9 | 5.6-49.2 | 4.5 | 2.4-6.1 |
| SDSC (0-1) | 0.87 | 0.70-0.96 | 0.63 | 0.39-0.93 | 0.95 | 0.81-0.99 |
| DSC (0-1) | 0.56 | 0.47-0.76 | 0.58 | 0.37-0.81 | 0.76 | 0.63-0.81 |
| HD98% (mm) | 6.7 | 4.2-90.3 | 18.1 | 4.6-83.2 | 5.7 | 3.0-6.0 |

**Case 3**

68-year old male, with a history of a cT3N0 rectal cancer for which he was treated with 5x5Gy and a LAR (ypT2N1, R1). Patient was diagnosed with LRRC and has received induction chemotherapy (3 cycles of CAPOX). Patient has been referred to you for chemo reirradiation and is planned for an APR with IORT.

Imaging:

- *Sigmoidoscopy at baseline:* No mucosal lesions
- *Baseline MRI:* Focal soft tissue at the anastomosis with a direct relationship to the sacrospinal/sacrotuberal ligament and the presacral fascia, maximal diameter of 2.5cm.
- *Baseline PET/CT:* Suspicion of an anastomotic recurrence.
- *MRI after induction chemotherapy:* No clear response to induction chemotherapy on the right sight. At the left, it is hard to differentiate between rest fibrosis and malignancy.
- *PET/CT after induction chemotherapy:* Anastomosis is no longer metabolically active.

**
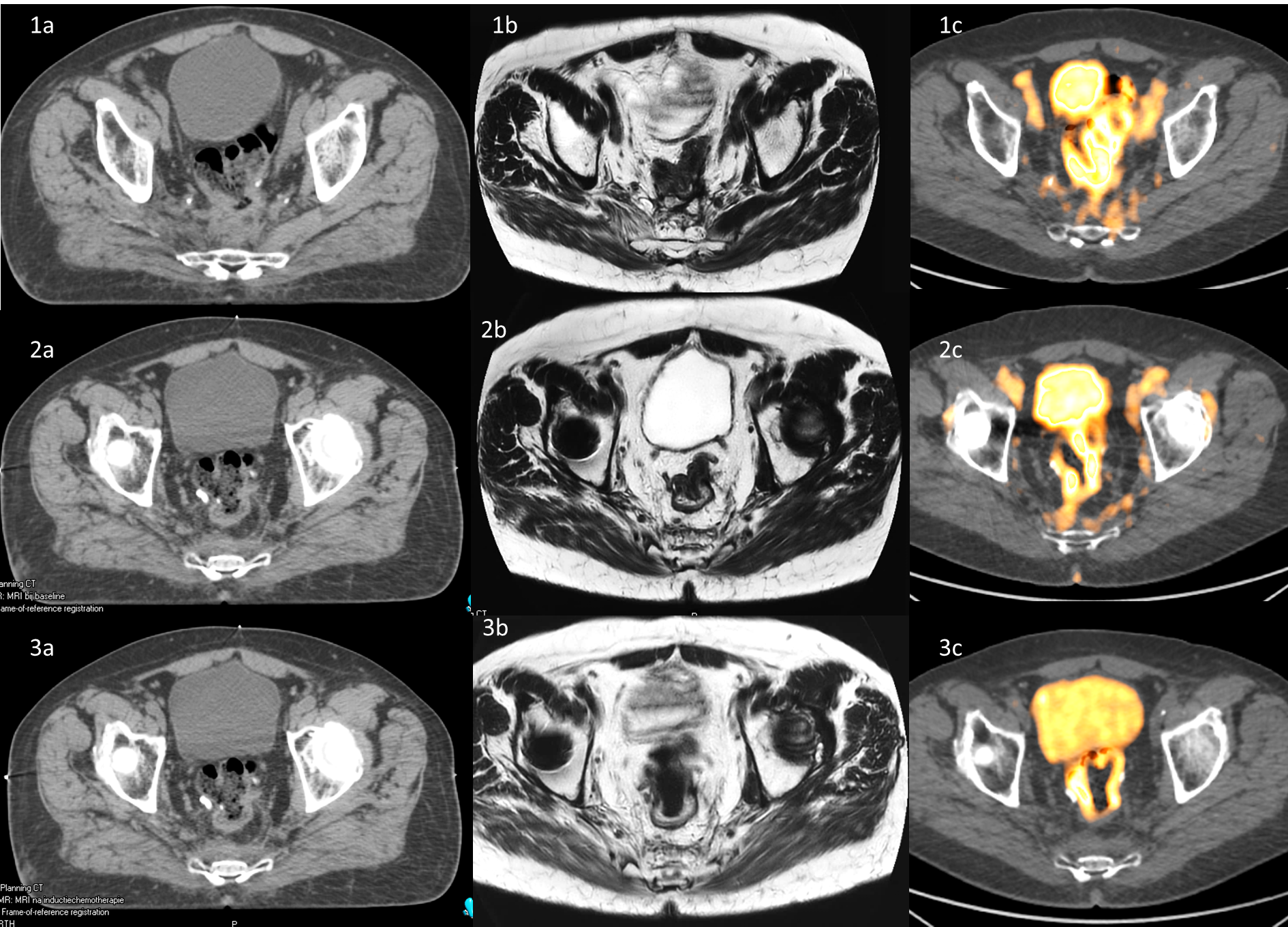
**

*Baseline (1, 2) planning CT (a), MRI (b), PET/CT (c), (3) Imaging following induction chemotherapy.*

**
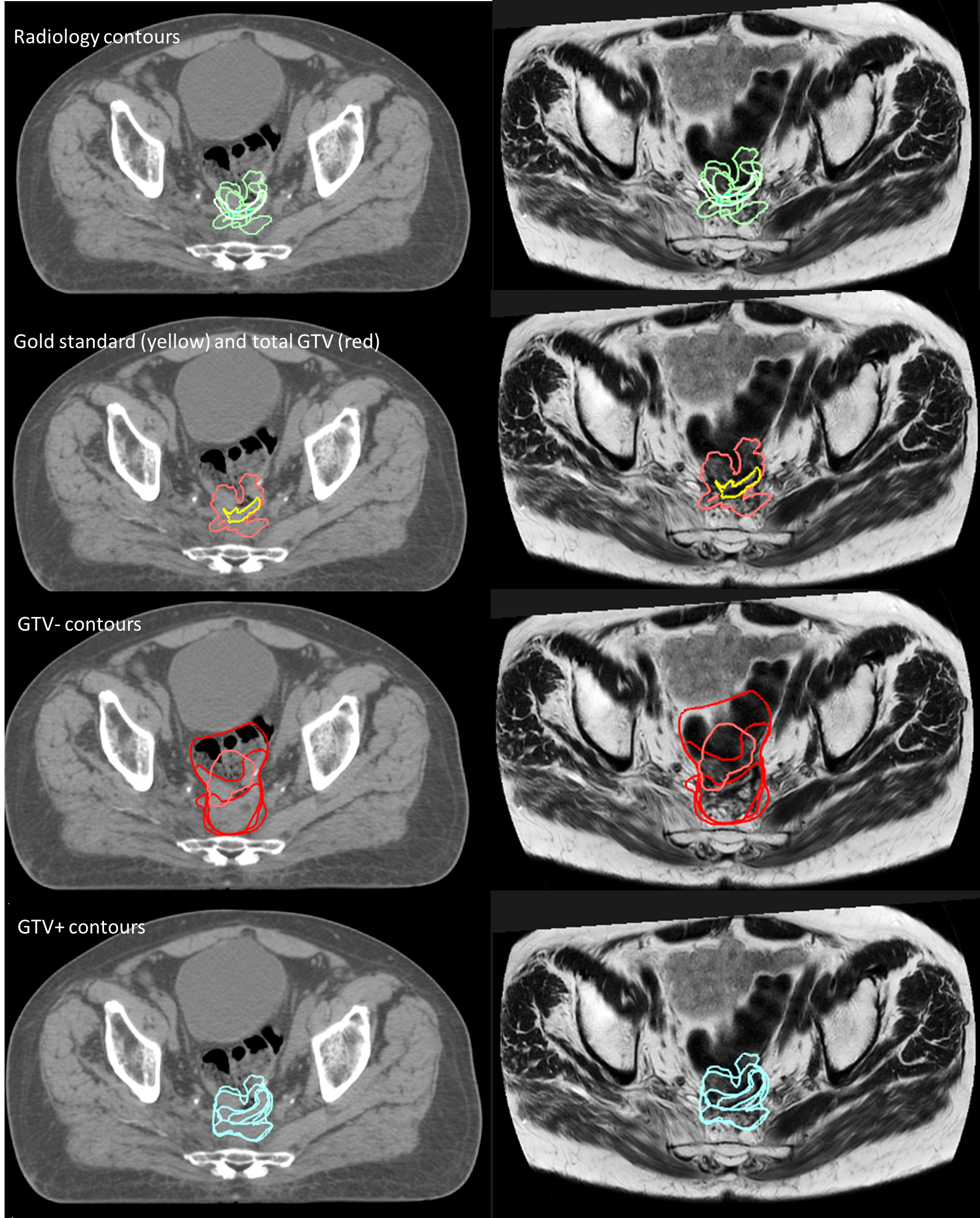
**

| **Case 3** | **RAD (n=8)** | | **GTV- (n=6)** | | **GTV+ (n=5)** | |
| --- | --- | --- | --- | --- | --- | --- |
|  | **Median** | **Range** | **Median** | **Range** | **Median** | **Range** |
| Volume (cc) | 13.9 | 1.2-38.7 | 59.5 | 11.0-219.2 | 27.4 | 3.3-93.4 |
| SDSC (0-1) | 0.56 | 0.21-0.70 | 0.42 | 0.26-0.87 | 0.63 | 0.25-0.92 |
| DSC (0-1) | 0.42 | 0.09-0.55 | 0.49 | 0.17-0.83 | 0.63 | 0.15-0.82 |
| HD98% (mm) | 24.3 | 14.1-51.9 | 34.5 | 7.5-45.0 | 27.5 | 6.0-51.2 |

**Case 4**

71-year old male with a history of a cT3N2M1 (liver) distal rectal cancer, for which he was treated with neoadjuvant CRT, 3 cycles of CAPOX-bevacizumab followed by resection of 5 liver metastases and a LAR (R0). Patient was diagnosed with a multifocal local recurrence for which he was planned to receive 4 cycles of induction chemotherapy (FOLFIRI-Bevacizumab), however due to toxicity only 1 cycle was given. Patient has been referred for chemo reirradiation. Patient is planned for a total pelvic exenteration with IORT.

Imaging:

- *Baseline MRI:* Suspicion of a multifocal recurrence in the lesser pelvis. Multiple nodular depositions on the mesorectal fascia, left and right in the lesser pelvis. The largest deposition is +/- 2cm. 2 additional possibly malignant lymph nodes.
- *Baseline PET/CT:* FDG-avid soft tissue in the pelvis, as on MRI, very suspicious for local recurrence. One FDG-avid presacral lymph node (on the right).
- *MRI after chemotherapy:* No response to therapy, stable disease.
- *PET-CT after chemotherapy:* no difference in PET-positive lymph nodes or local multifocal recurrence. No distant metastases.


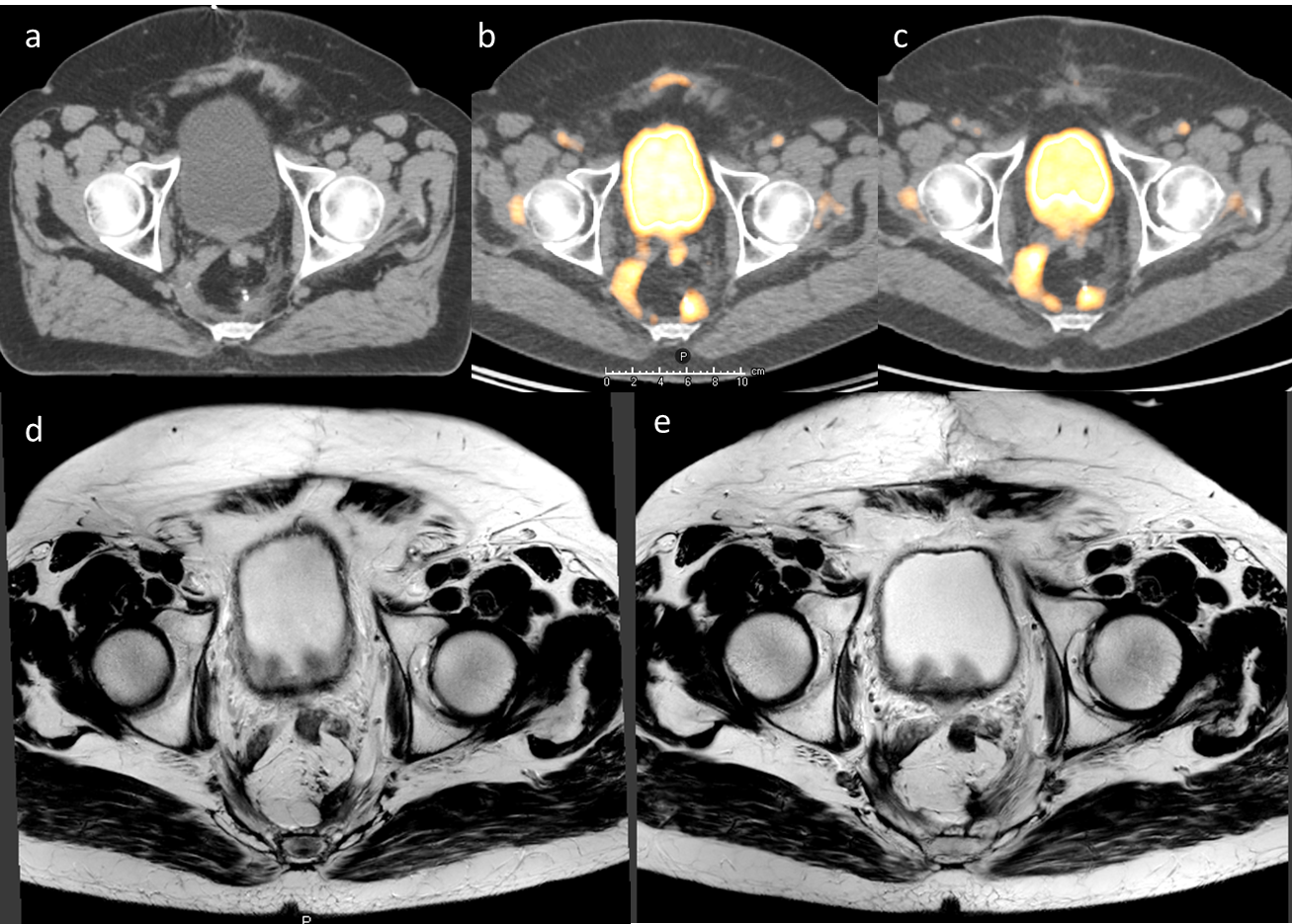


*Figure showing: (a) planning CT (b) PET-CT at baseline (c) PET-CT after chemotherapy (d) Baseline MRI (e) MRI after induction chemotherapy*


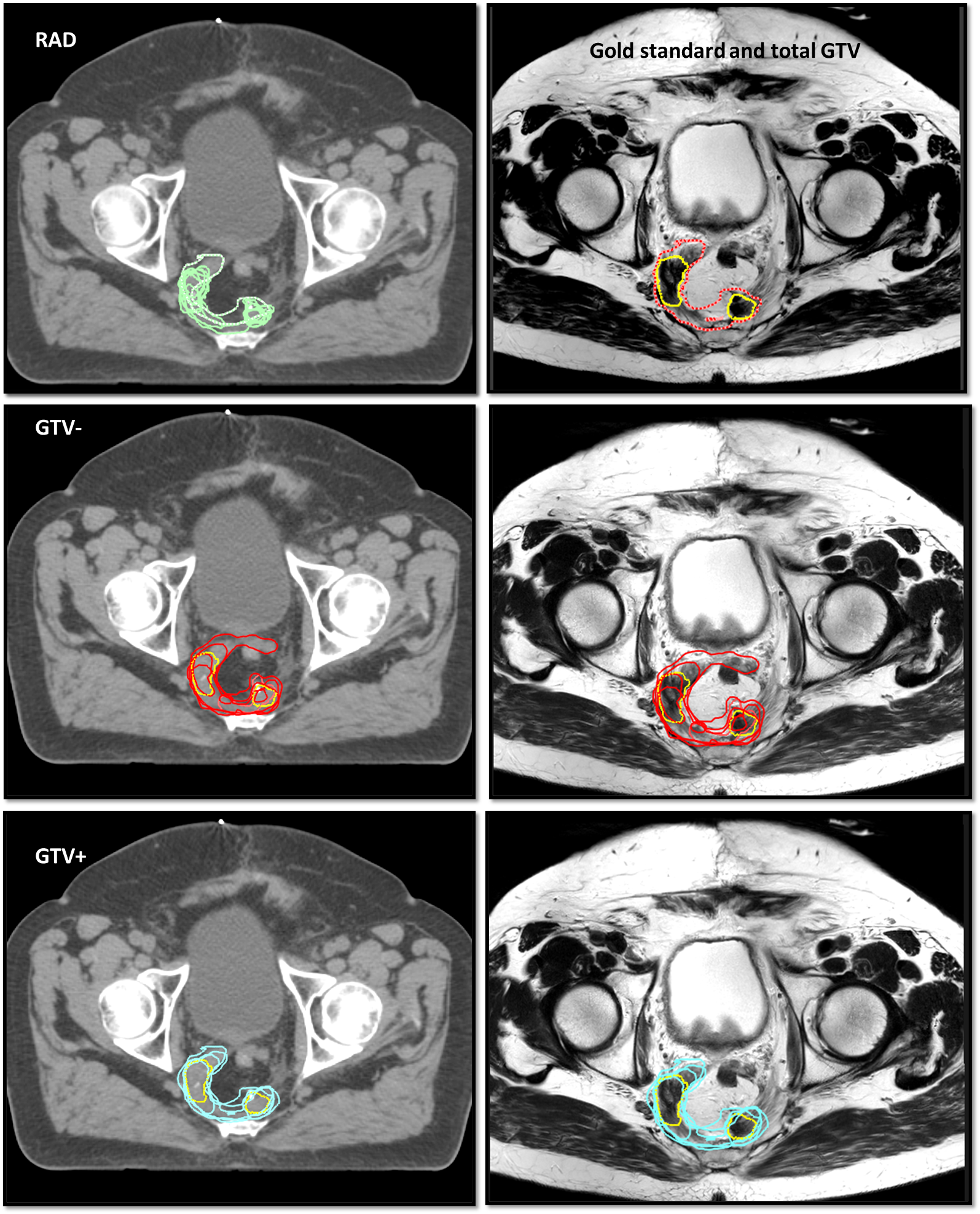


| **Case 4** | **RAD (n=8)** | | **GTV- (n=5)** | | **GTV+ (n=4)** | |
| --- | --- | --- | --- | --- | --- | --- |
|  | **Median** | **Range** | **Median** | **Range** | **Median** | **Range** |
| Volume (cc) | 34.2 | 17.6-63.9 | 83.0 | 36.9-121.1 | 91.3 | 72.8-114.7 |
| SDSC (0-1) | 0.80 | 0.61-0.87 | 0.75 | 0.68-0.86 | 0.88 | 0.72-0.91 |
| DSC (0-1) | 0.60 | 0.45-0.71 | 0.69 | 0.62-0.82 | 0.82 | 0.68-0.85 |
| HD98% (mm) | 17.4 | 7.0-68.5 | 19.0 | 9.0-25.5 | 15.3 | 7.3-60.3 |

**Case 5**

41-year old male, with a history of a T3N0 distal rectal cancer for which an APR was performed (pT3N1, R1). Patient presented with a multifocal recurrence and has been referred for neoadjuvant CRT. Patient is planned for surgery with IORT.

Imaging:

- *Baseline MRI:* 2 presacral lesions of approximately 8mm, probably pathological lymph nodes.
- *Baseline PET-CT:* PET-positive area left ventrolaterally of the coccygeal bone. The second PET-positive lesion is situated dorsally of the prostate. Both are suspicious for a local recurrence. 2 slightly PET-positive presacral lymph nodes, which could be fitting with the MRI findings? No metastases elsewhere.


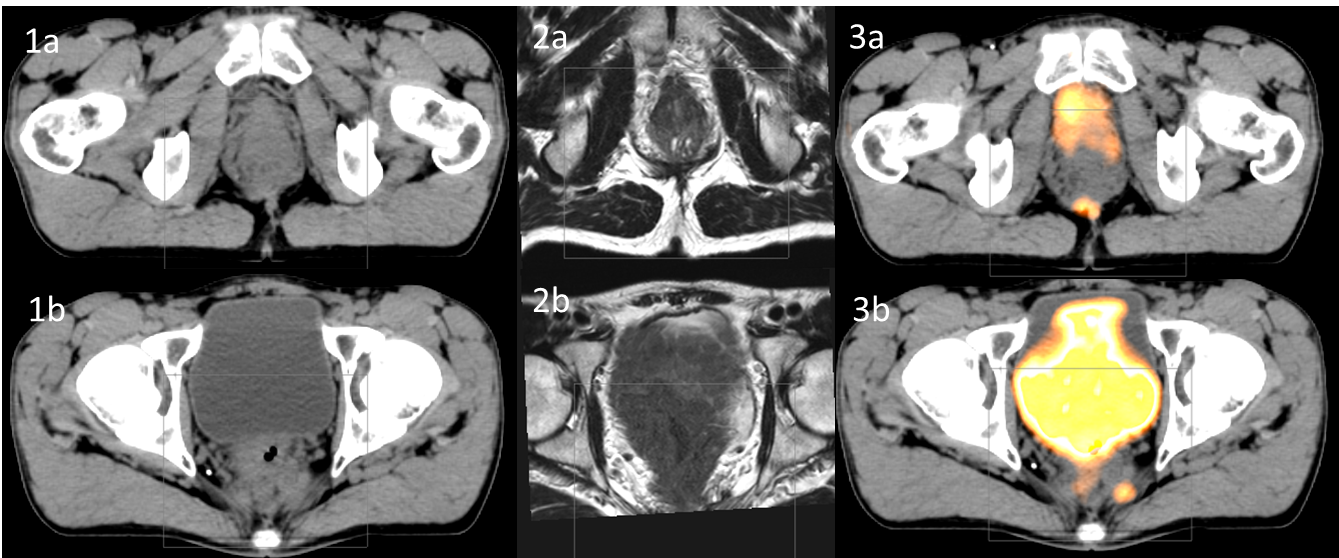


*Figure showing (1a+b) planning CT, (2a+b) baseline MRI and (3a+b) baseline PET-CT*.


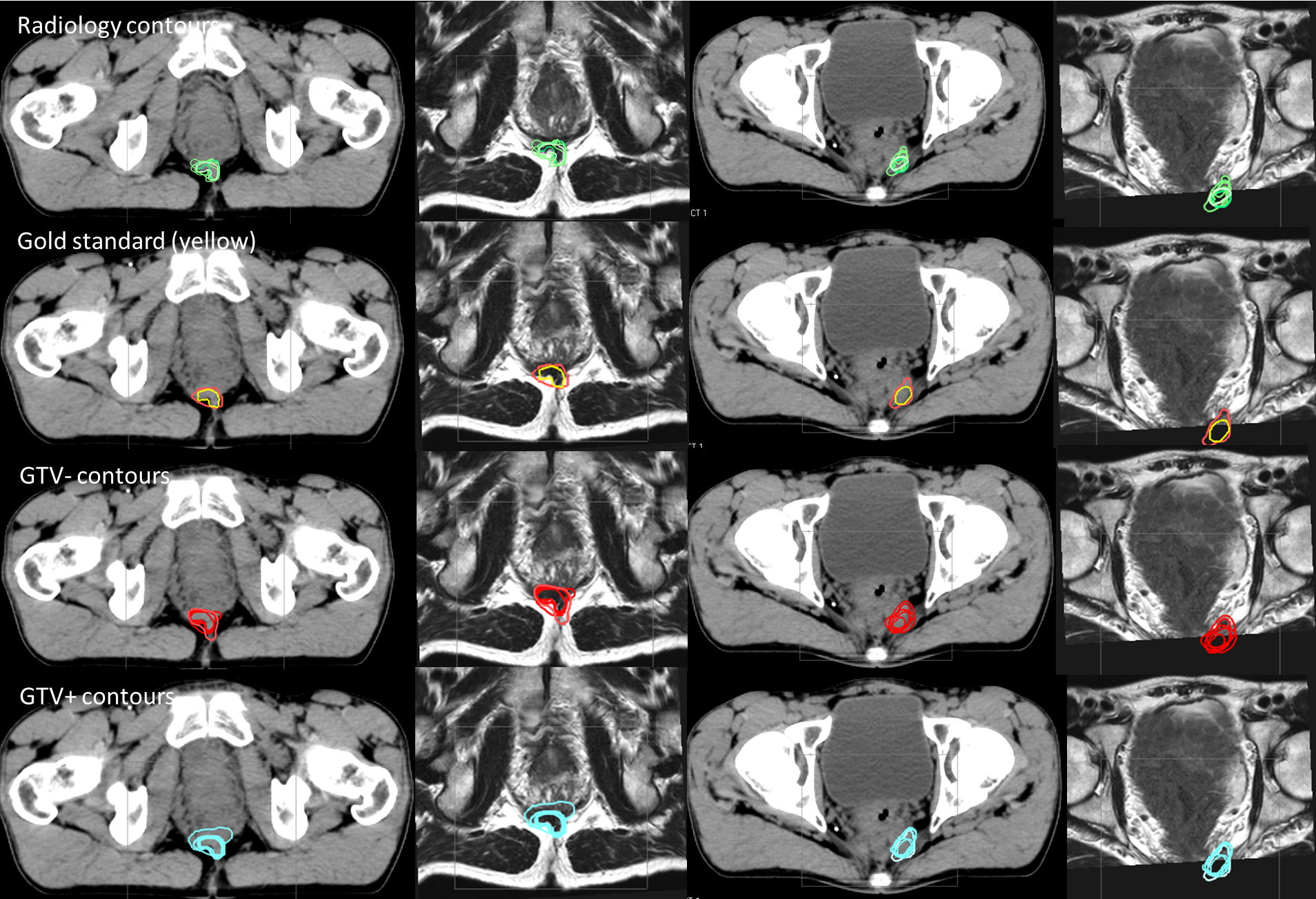


| **Case 5** | **RAD (n=8)** | | **GTV- (n=5)** | | **GTV+ (n=6)** | |
| --- | --- | --- | --- | --- | --- | --- |
|  | **Median** | **Range** | **Median** | **Range** | **Median** | **Range** |
| Volume (cc) | 5.6 | 3.6-11.6 | 10.7 | 6.8-24.2 | 11.6 | 7.2-16.8 |
| SDSC (0-1) | 0.86 | 0.65-0.91 | 0.87 | 0.65-0.96 | 0.91 | 0.77-0.99 |
| DSC (0-1) | 0.62 | 0.55-0.74 | 0.74 | 0.56-0.76 | 0.76 | 0.61-0.85 |
| HD98% (mm) | 31.1 | 6.1-48.8 | 36.6 | 3.5-44.5 | 7.8 | 3.0-15.6 |

**Case 6**

78-year old male, with a history of a T3N1 rectosigmoidal tumour for which a LAR was performed. Patient is presenting with an anastomotic recurrence and was referred for full-course CRT. Patient is planned for a re-LAR with IORT.

Imaging:

- *Colonoscopy at baseline:* suspicious lesion on the anastomosis in the rectum. PA: adenocarcinoma.
- *MRI at baseline:* Anastomotic recurrence presacral extending towards the sacrum, without evidence of involvement of the sacrum. Also suspicion of a nodal recurrence at the right. Central, right lateral and posterior compartment involved.
- *PET-CT at baseline:* No distant metastases. Prominent local and nodal recurrence (near v.a. iliaca right).


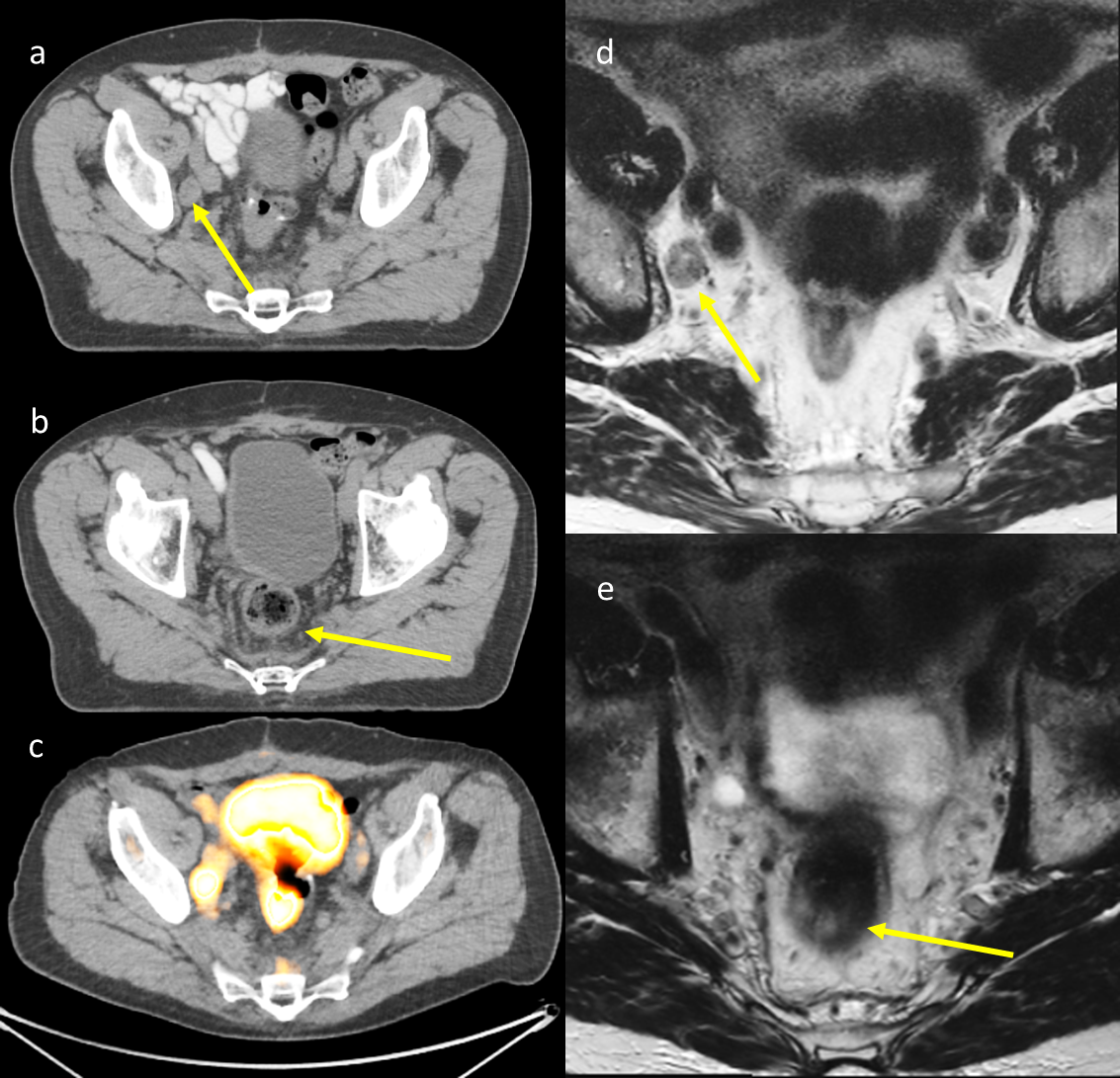


*Figure showing (a+d) nodal recurrence on planning CT and MRI (b+e) anastomotic recurrence (c) baseline PET/CT*


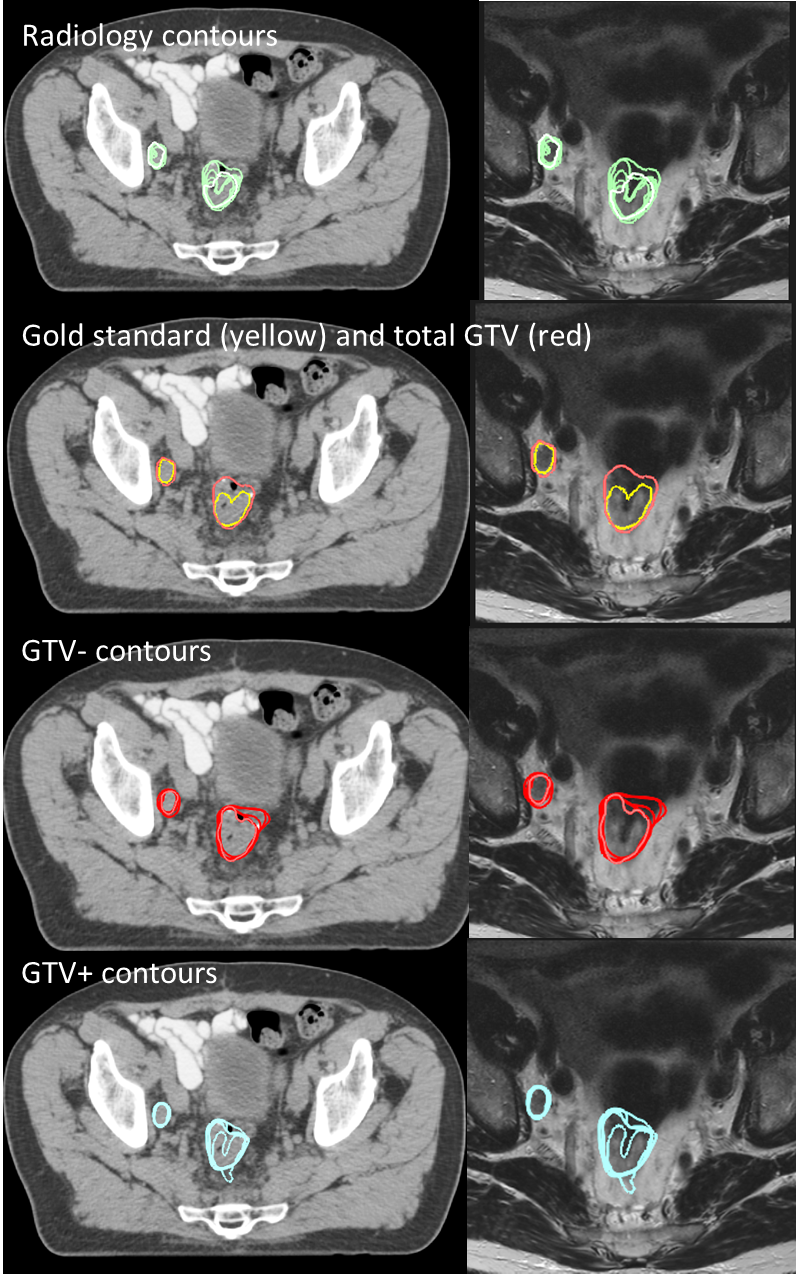


| **Case 6** | **RAD (n=8)** | | **GTV- (n=6)** | | **GTV+ (n=5)** | |
| --- | --- | --- | --- | --- | --- | --- |
|  | **Median** | **Range** | **Median** | **Range** | **Median** | **Range** |
| Volume (cc) | 17.1 | 4.7-22.9 | 29.8 | 23.9-45.0 | 20.0 | 15.7-32.4 |
| SDSC (0-1) | 0.84 | 0.47-0.98 | 0.86 | 0.73-0.95 | 0.89 | 0.77-0.99 |
| DSC (0-1) | 0.72 | 0.40-0.84 | 0.80 | 0.73-0.82 | 0.77 | 0.75-0.91 |
| HD98% (mm) | 7.5 | 3.5-26.8 | 12.5 | 4.0-15.3 | 6.6 | 3.0-11.4 |

**Case 7**

63-year old male, with a history of a Locally advanced rectal cancer (cTNM stage unknown), treated with CRT and an APR (ypT1N0, R0). Patient was diagnosed with a nodal recurrence and has received induction chemotherapy (3x CAPOX). Patient has been referred for reirradiation and is planned for surgery with IORT.

Imaging:

- *MRI at baseline:* Suspicion of a lateral nodal recurrence (left) with a diameter of 1.7cm, in contact with the internal iliac artery.
- *PET/CT at baseline:* FDG-avid focus laterally against the mesorectal fascia left. No distant metastases.
- *PET/CT after inductionchemotherapy:* Strong regression of FDG-uptake in the node against the internal iliac artery left. Only a small amount of activity left. No new lesions.

*
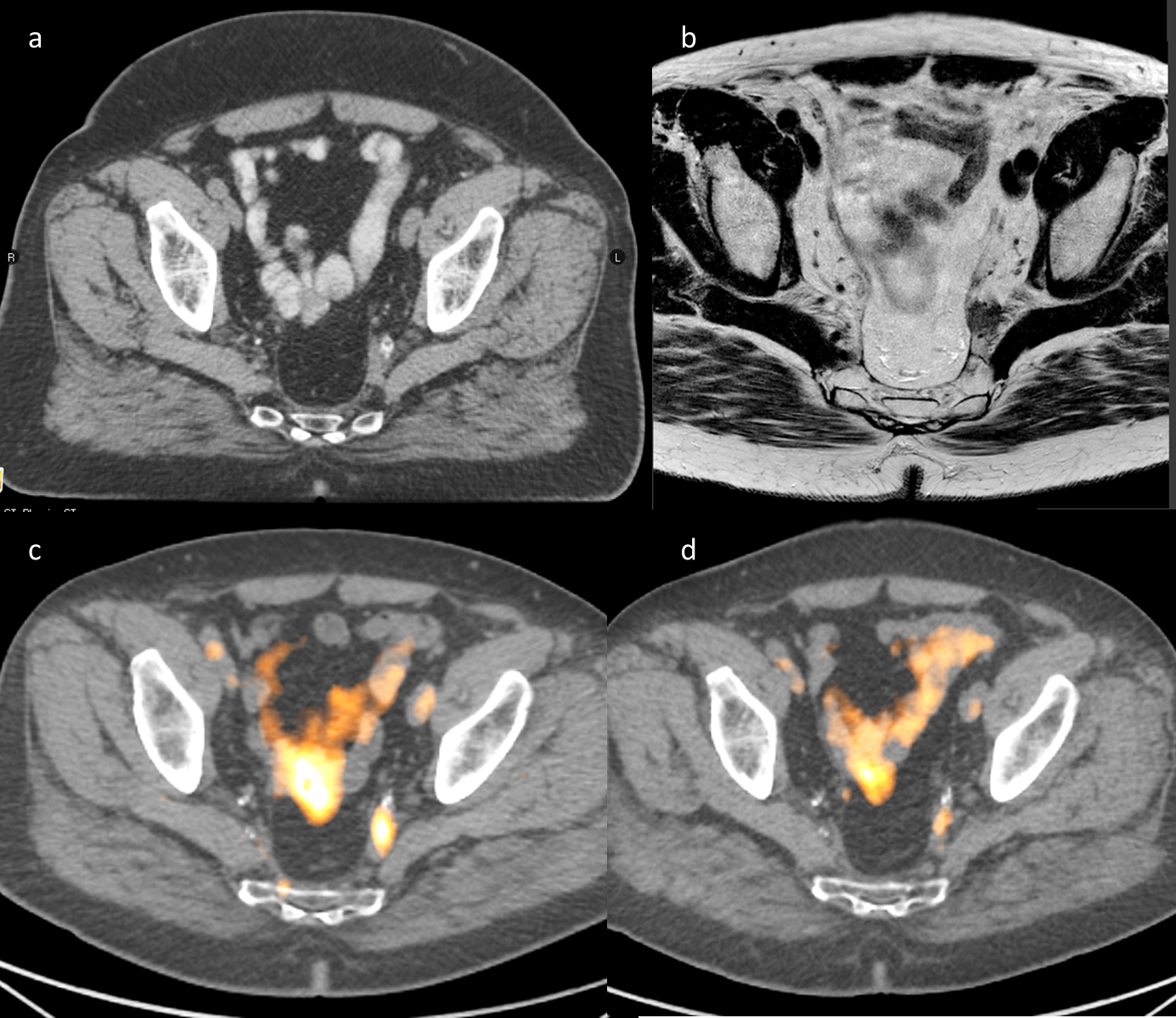
*

*Figure showing (a) planning CT, (b) baseline MRI (c) PET/CT at baseline (c) Pet-CT after induction chemotherapy*

| **Case 7** | **RAD (n=8)** | | **GTV- (n=6)** | | **GTV+ (n=5)** | |
| --- | --- | --- | --- | --- | --- | --- |
|  | **Median** | **Range** | **Median** | **Range** | **Median** | **Range** |
| Volume (cc) | 1.7 | 0.98-39.9 | 4.6 | 2.5-10.4 | 4.5 | 1.9-7.9 |
| SDSC (0-1) | 0.32 | 0.00-0.81 | 0.85 | 0.75-0.94 | 0.92 | 0.50-0.95 |
| DSC (0-1) | 0.17 | 0.00-0.48 | 0.71 | 0.58-0.80 | 0.74 | 0.40-0.84 |
| HD98% (mm) | 22.0 | 6.4-186.5 | 7.0 | 6.0-11.7 | 5.8 | 4.2-39.0 |

*
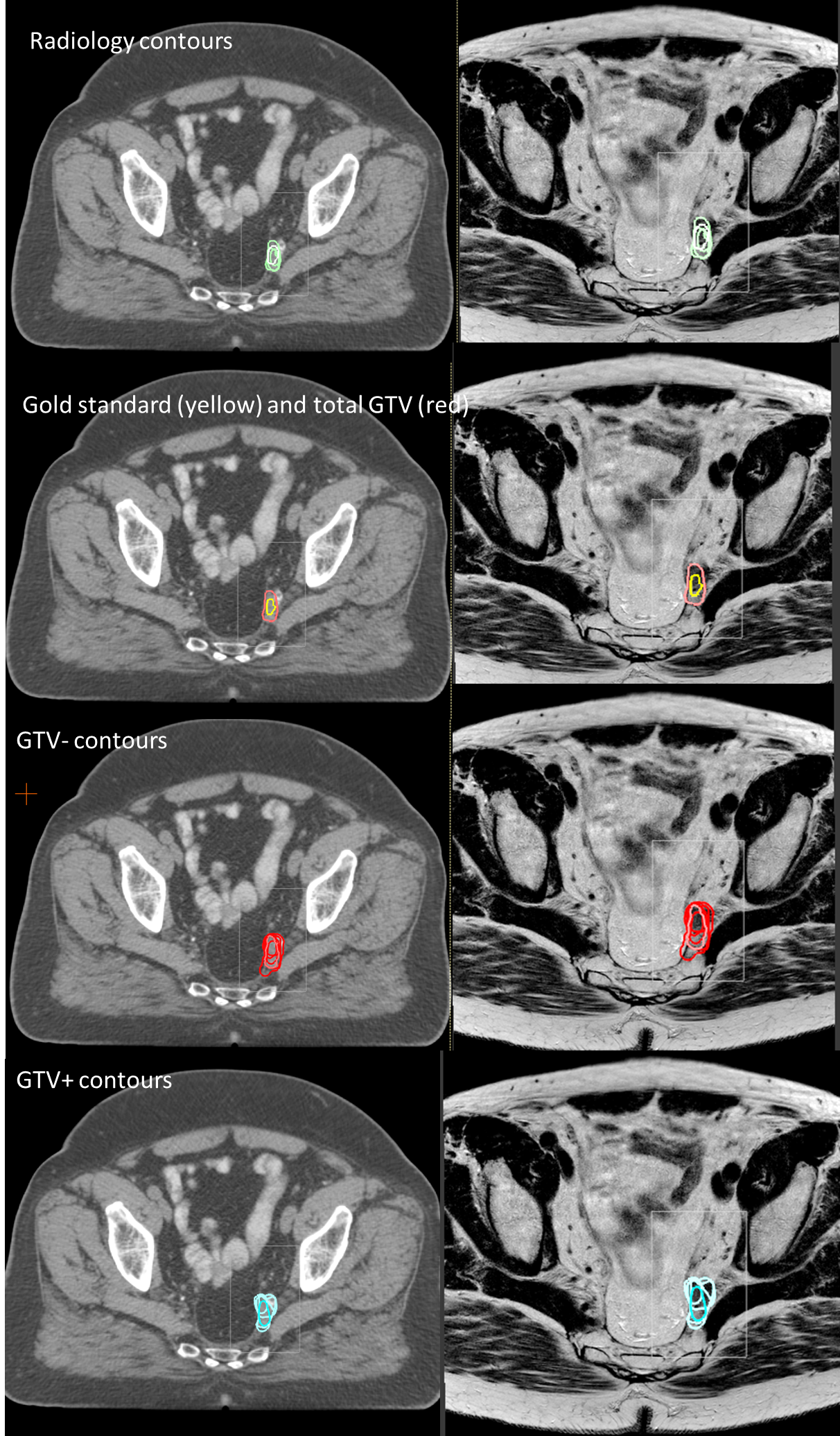
*

**Case 8**

68-year old male, presenting with a dorsolateral LRRC after a primary rectal cancer (cTNM stage unknown), treated by 5x5Gy radiotherapy and an APR (pT2N1, R0). Patient is referred after induction chemotherapy (3 cycles of CAPOX). Following chemo reirradiation, patient is planned for surgery with intra-operative radiotherapy on the lateral pelvic wall.

Imaging:

- Baseline MRI: Suspicion of a unifocal local recurrence against the right pelvic side wall.
- Baseline PET-CT: No suspicious lesions outside of the pelvis. Increased FDG uptake can be seen at the right pelvic side wall.
- *PET-CT following induction chemotherapy:* Good metabolic response, possible complete response. No new lesions on PET-CT.


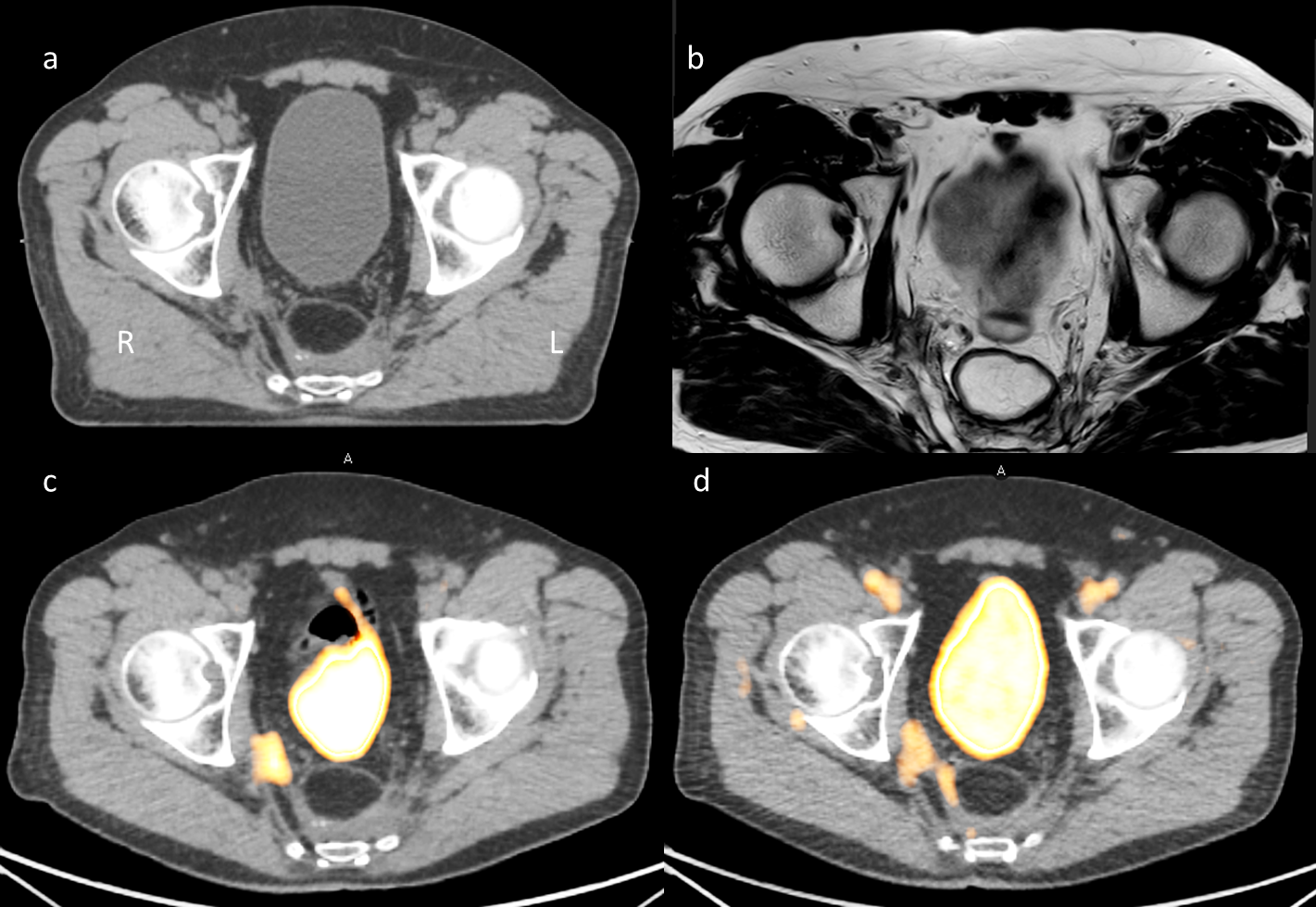


Figure showing (a) Planning CT, (b) MRI at baseline (c) PET-CT prior to induction chemotherapy (d) PET-CT following induction chemotherapy


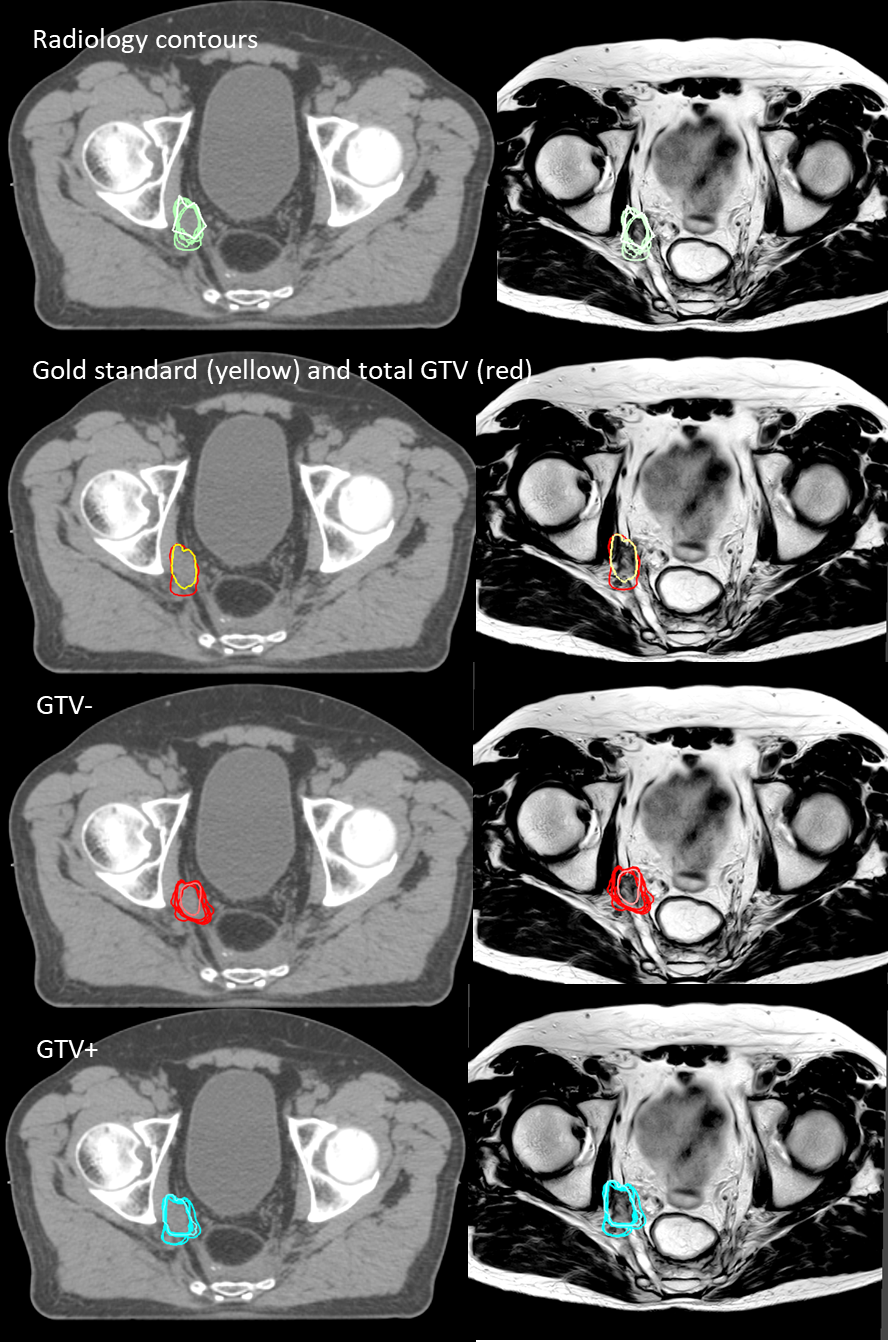


| **Case 8** | **RAD (n=8)** | | **GTV- (n=6)** | | **GTV+ (n=6)** | |
| --- | --- | --- | --- | --- | --- | --- |
|  | **Median** | **Range** | **Median** | **Range** | **Median** | **Range** |
| Volume (cc) | 6.0 | 3.8-8.1 | 9.1 | 5.1-9.9 | 10.2 | 7.0-11.7 |
| SDSC (0-1) | 0.94 | 0.87-0.98 | 0.92 | 0.65-0.96 | 0.94 | 0.88-0.97 |
| DSC (0-1) | 0.78 | 0.68-0.81 | 0.84 | 0.64-0.85 | 0.84 | 0.75-0.86 |
| HD98% (mm) | 5.8 | 3.2-8.5 | 6.1 | 6.0-9.4 | 5.1 | 3.8-6.0 |

**Case 9**

70-year old female with a history of a LARC (unknown cTNM) for which she received CRT and a LAR (pT2N1).

Patient was diagnosed with a distal LRRC. Patient has received 3 cycles of CAPOX, and is now referred for chemo reirradiation. Patient will undergo debulking surgery with IORT.

Imaging:

- *Colonoscopy at baseline:* Suspicion of a local recurrence distally in the rectum. PA: Adenocarcinoma.
- *MRI at baseline:* Anastomotic recurrence (+/- 2cm) with possible involvement of the cervix.
- *MRI after induction chemotherapy:* The local recurrence in the rectal stump is no longer visible. Fibrosis visible. >75% decrease in volume.

**
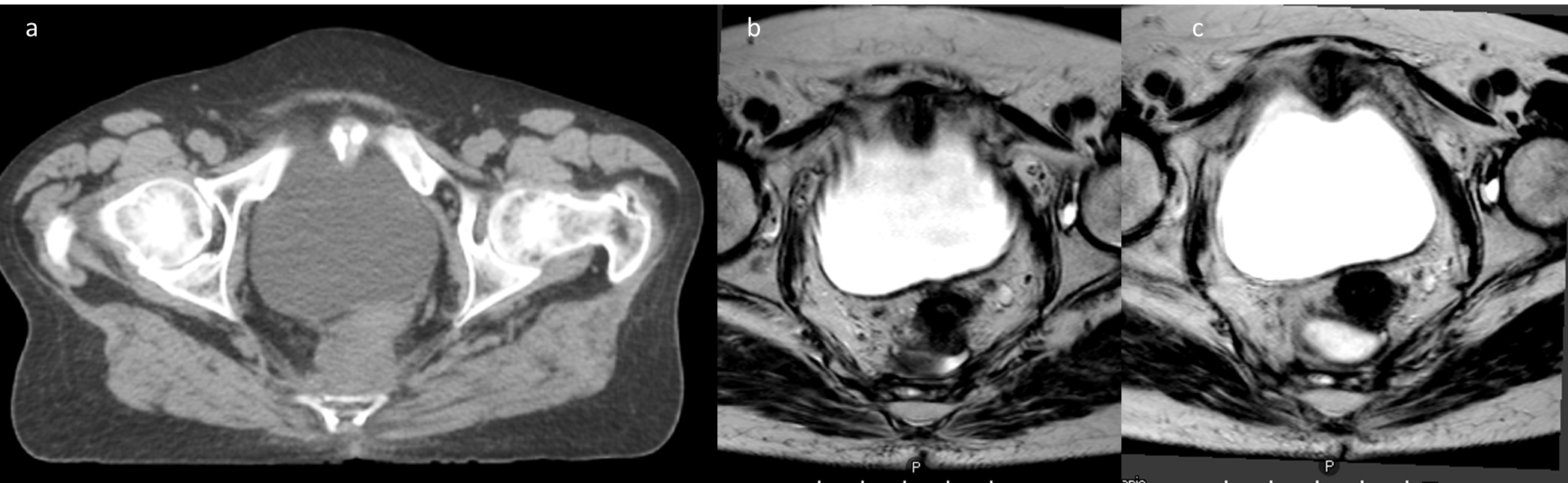
**

*Figure showing (a) planning CT (b) MRI at baseline (c) MRI after induction chemotherapy*

| **Case 9** | **RAD (n=8)** | | **GTV- (n=6)** | | **GTV+ (n=5)** | |
| --- | --- | --- | --- | --- | --- | --- |
|  | **Median** | **Range** | **Median** | **Range** | **Median** | **Range** |
| Volume (cc) | 4.2 | 1.6-7.1 | 11 | 8.5-30.3 | 19.3 | 3.7-28.7 |
| SDSC (0-1) | 0.86 | 0.02-0.97 | 0.71 | 0.56-0.92 | 0.71 | 0.34-0.89 |
| DSC (0-1) | 0.63 | 0.00-0.77 | 0.64 | 0.50-0.79 | 0.69 | 0.27-0.81 |
| HD98% (mm) | 7.5 | 3.3-26.7 | 12.1 | 8.2-14.7 | 12.0 | 8.2-21.0 |

**
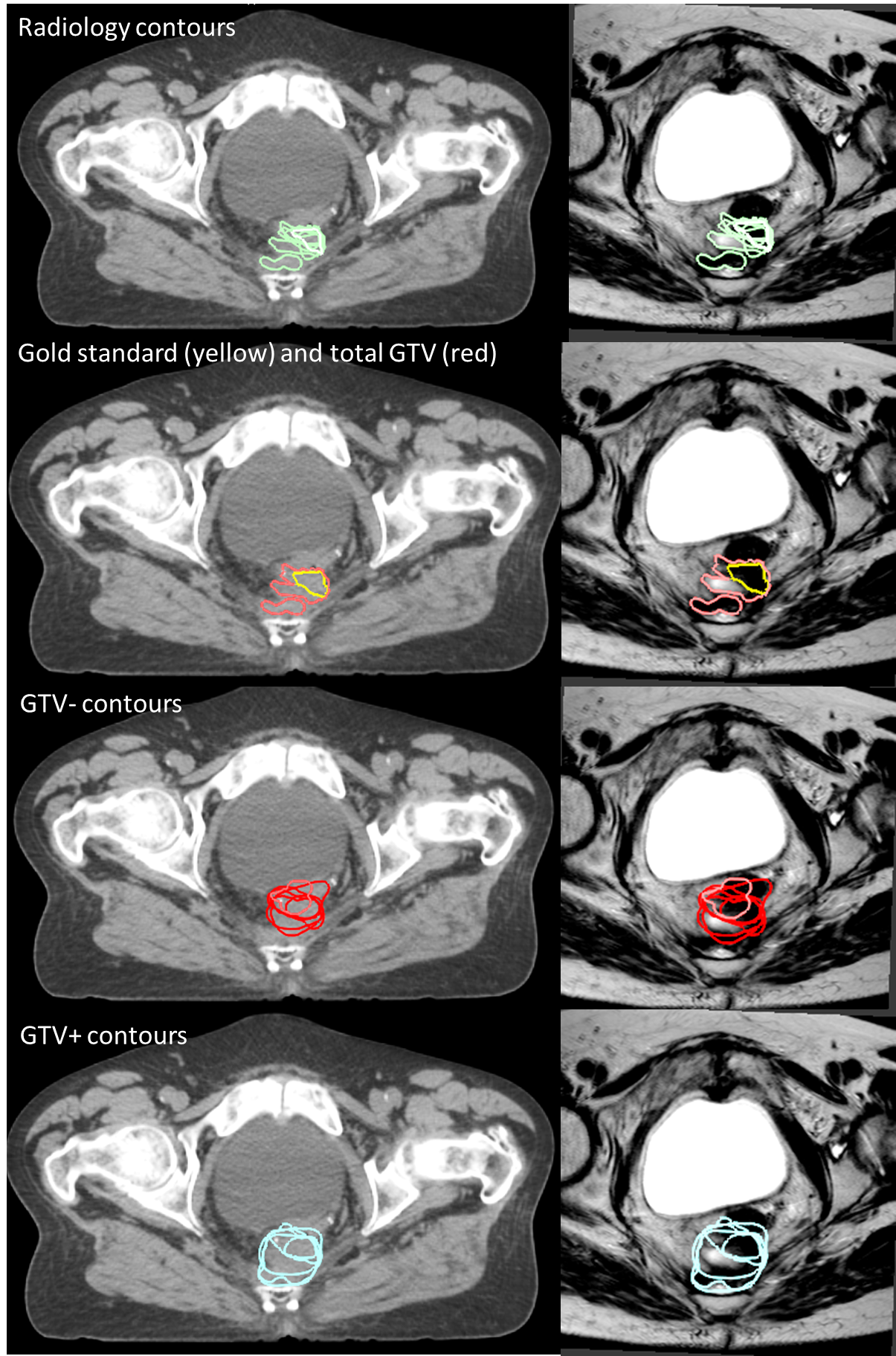
**

**Case 10**

65-year old female, with a history of a cT3N1 (MRF+) rectal cancer for which she was treated with induction chemotherapy (4 cycles of CAPOX), full-course CRT and an APR (ypT2N0, R0). Patient presented with a solitary nodal recurrence and has been referred for chemo reirradiation. Patient is planned for a surgical resection with IORT.

Imaging:

- *Baseline MRI:* Solid nodule in the lesser pelvis on the left, approximately 1.2cm, located in the obturator loge.
- *Baseline PET/CT:* Hypermetabolic lymph node dorsally in the obturator loge left, suspicious for a solitary lymph node metastasis.


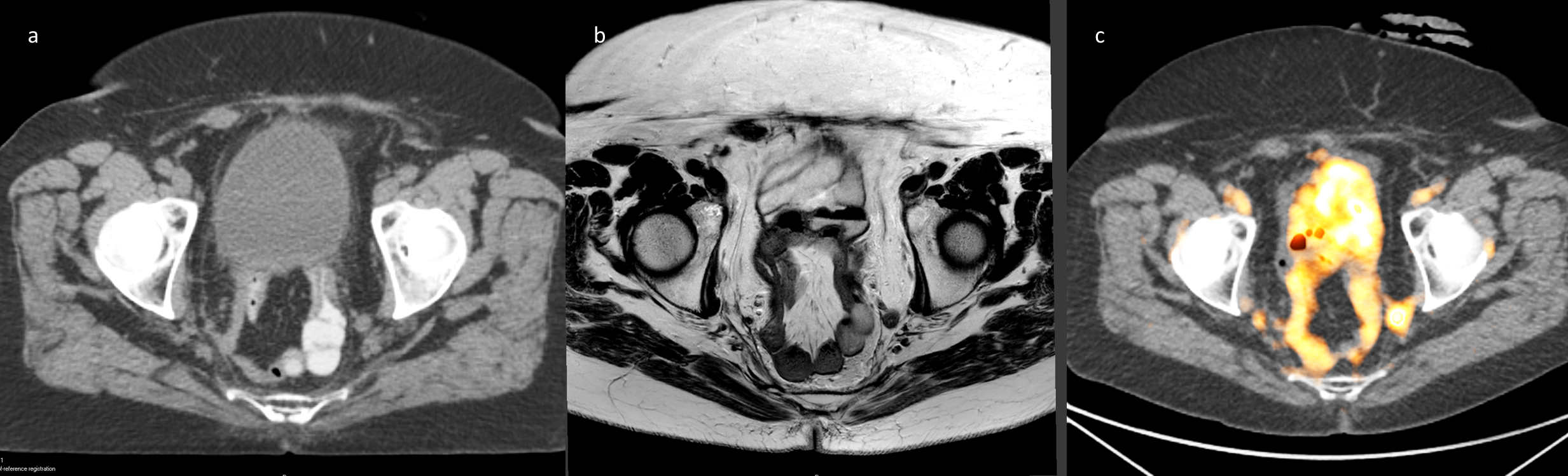


Figure showing (a) planning CT (b) MRI at baseline (c) PET/CT at baseline

| **Case 10** | **RAD (n=8)** | | **GTV- (n=5)** | | **GTV+ (n=5)** | |
| --- | --- | --- | --- | --- | --- | --- |
|  | **Median** | **Range** | **Median** | **Range** | **Median** | **Range** |
| Volume (cc) | 1.0 | 0.8-1.4 | 1.4 | 1.0-2.5 | 1.5 | 1.2-2.2 |
| SDSC (0-1) | 0.99 | 0.83-1.00 | 1.00 | 0.98-1.00 | 1.00 | 0.97-1.00 |
| DSC (0-1) | 0.76 | 0.47-0.85 | 0.81 | 0.68-0.84 | 0.81 | 0.77-0.82 |
| HD98% (mm) | 3.1 | 3.0-5.0 | 2.3 | 2.3-3.2 | 3.0 | 3.0-6.0 |


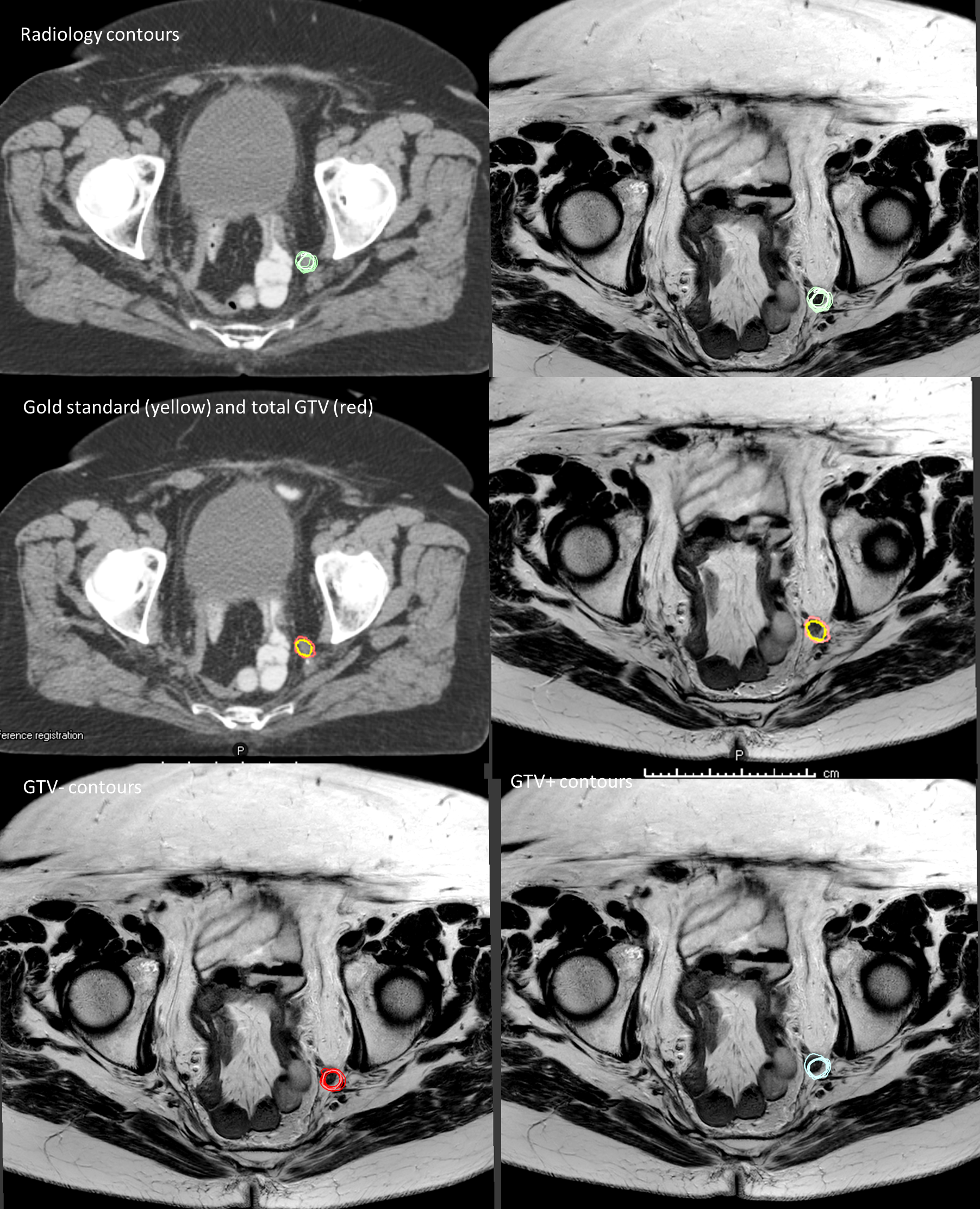


**Case 11**

73-year old male, with a history of a cT3bN0 (MRF+) distal rectal cancer for which he was treated with CRT and a LAR (TaTME, ypT3N1, R0). Patient has a presacral local recurrence and has received induction chemotherapy (3 cycles of CAPOX). He was referred for chemo reirradiation. Patient is planned for resection with IORT.

Imaging:

- *Baseline MRI:* suspicion of a unifocal presacral (maximal diameter approximately 26mm).
- *Baseline PET-CT:* High presacral uptake (S4), suspicion of a local recurrence. Two high uptake foci in the prostate, DD prostate carcinoma or focal prostatitis.
- *Additional prostate MRI:* diffuse low T2 signal in the peripheral zone on the right, as fitting for prostatitis. No suspicion of a prostate cancer (PI-RADS 2).
- *MRI after chemotherapy:* Known presacral recurrence with only minimal response. There is still a suspicion of vital tumour.
- *PET/CT after chemotherapy:* clear reduction of metabolic activity in the lesser pelvis at the known local recurrence. No distant metastases.


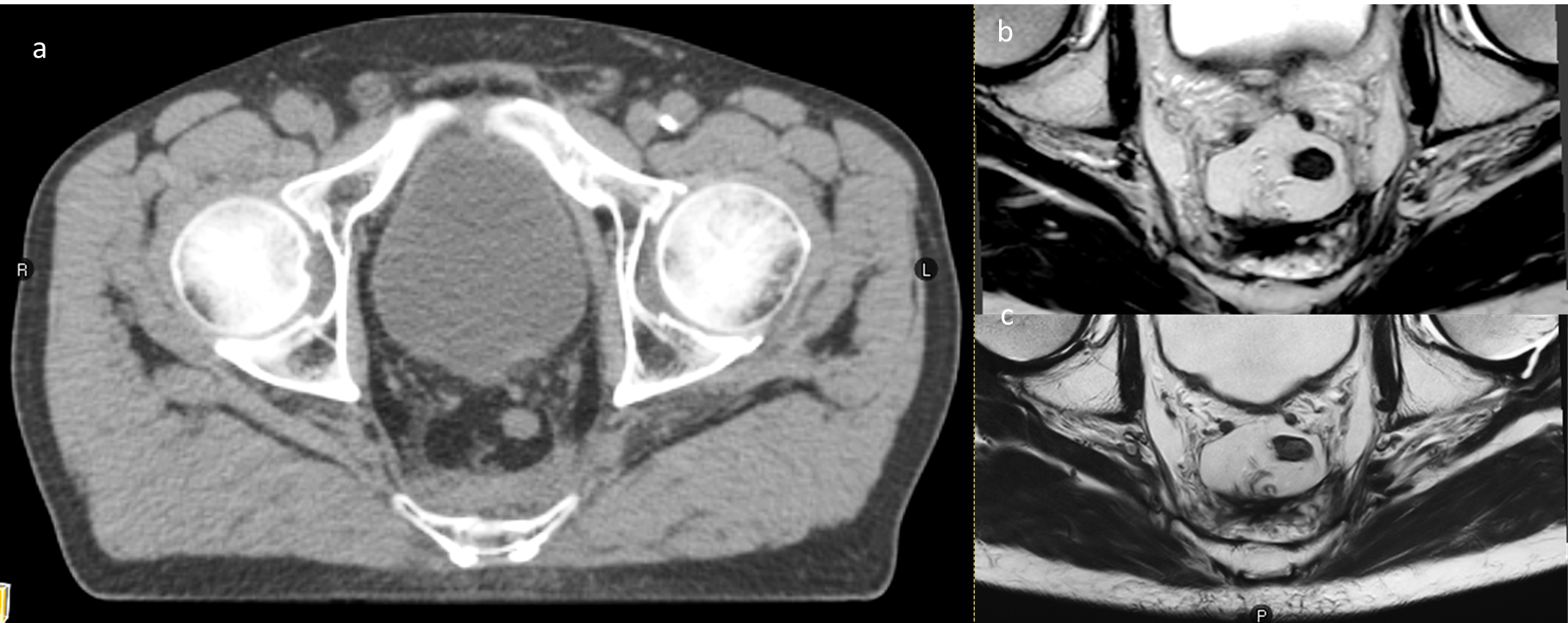


*Figure showing: (a) planning CT (b) Baseline MRI (c) MRI after induction chemotherapy*

| **Case 11** | **RAD (n=8)** | | **GTV- (n=6)** | | **GTV+ (n=5)** | |
| --- | --- | --- | --- | --- | --- | --- |
|  | **Median** | **Range** | **Median** | **Range** | **Median** | **Range** |
| Volume (cc) | 1.9 | 0.1-7.9 | 15.6 | 6.1-43.5 | 13.6 | 5.2-49.6 |
| SDSC (0-1) | 0.44 | 0.31-0.84 | 0.63 | 0.37-0.92 | 0.60 | 0.30-0.96 |
| DSC (0-1) | 0.32 | 0.10-0.69 | 0.65 | 0.46-0.84 | 0.65 | 0.42-0.87 |
| HD98% (mm) | 9.3 | 4.6-26.7 | 11.1 | 4.6-17.3 | 12.3 | 4.2-18.9 |


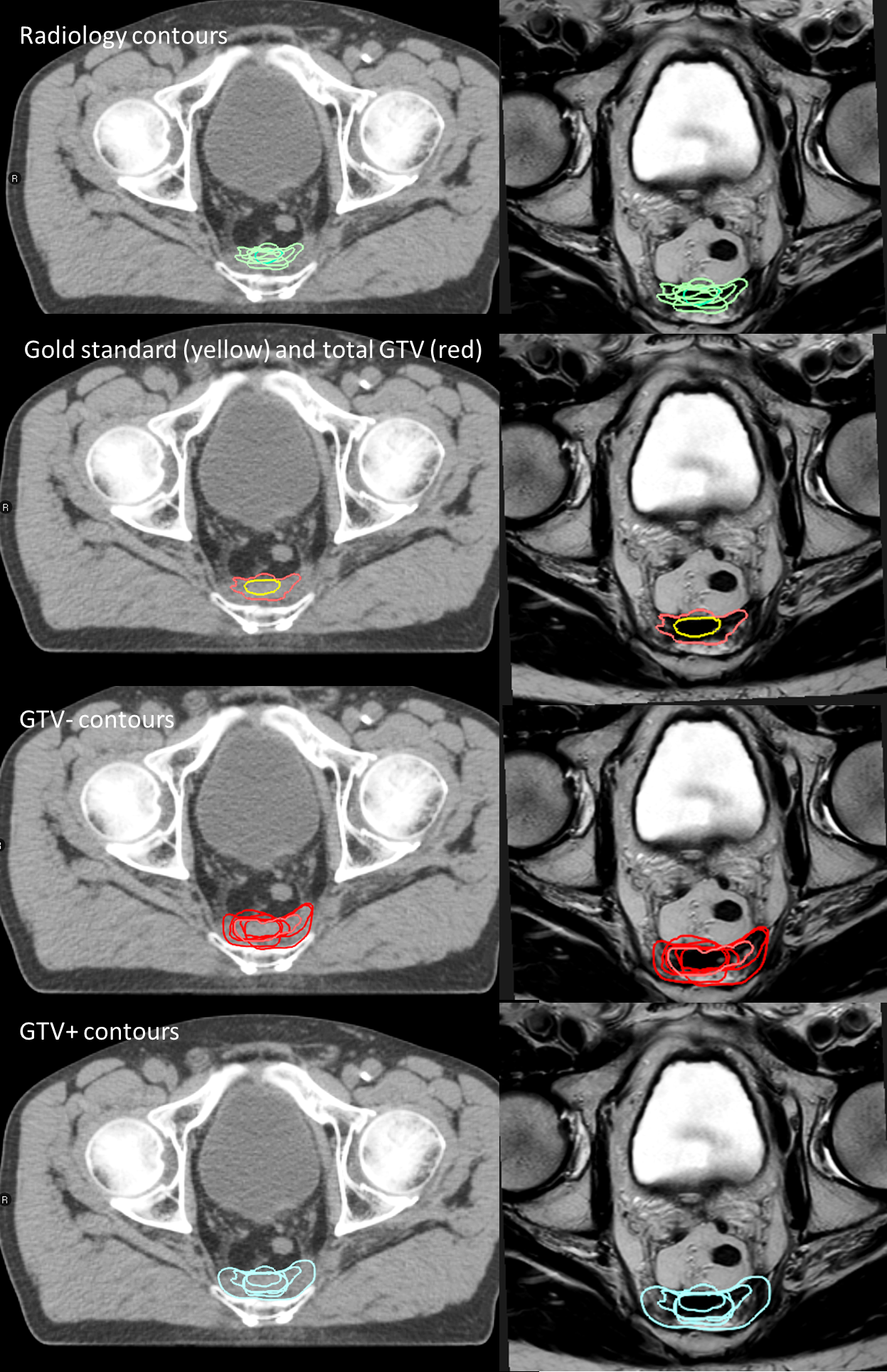


**Case 12**

68-year old male, presenting with a local recurrence within a large presacral abscess after primary rectal cancer nearly 20 years ago, treated with chemoradiotherapy (50.4Gy) and a low anterior resection. Patient has received 4 cycles of FOLFOX-bevacizumab and is now referred for chemo reirradiation. Patient is planned for surgical resection with IORT following reirradiation.

Imaging:

- *Baseline MRI:* Known presacral abscess. Inside the abscess is a large soft tissue mass of approximately 10cm up growing until the anus. Possible sacral involvement at S4.
- *Baseline PET-CT:* Intense FDG-uptake presacral. No evidence of distant disease or lymph node involvement.
- *MRI after induction chemotherapy:* Evident response after chemotherapy, possibly residual soft tissue mass at S3-S4.
- *PET-CT after induction chemotherapy:* Partial metabolic response of the local recurrence.


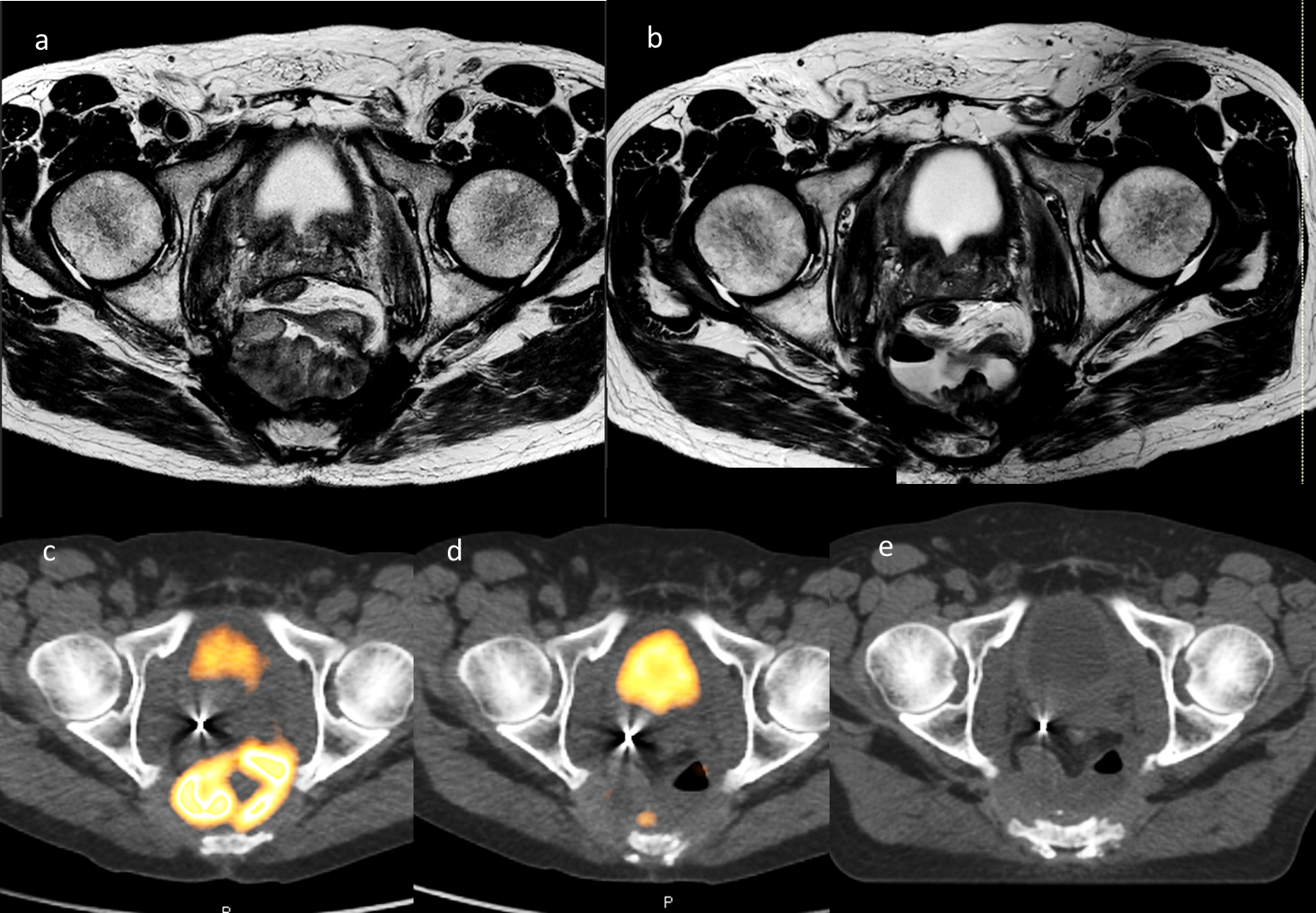


*Figure showing (a) baseline MRI (b) MRI after induction chemotherapy (c) baseline PET-CT (d) PET-CT after induction chemotherapy (e) planning CT.*


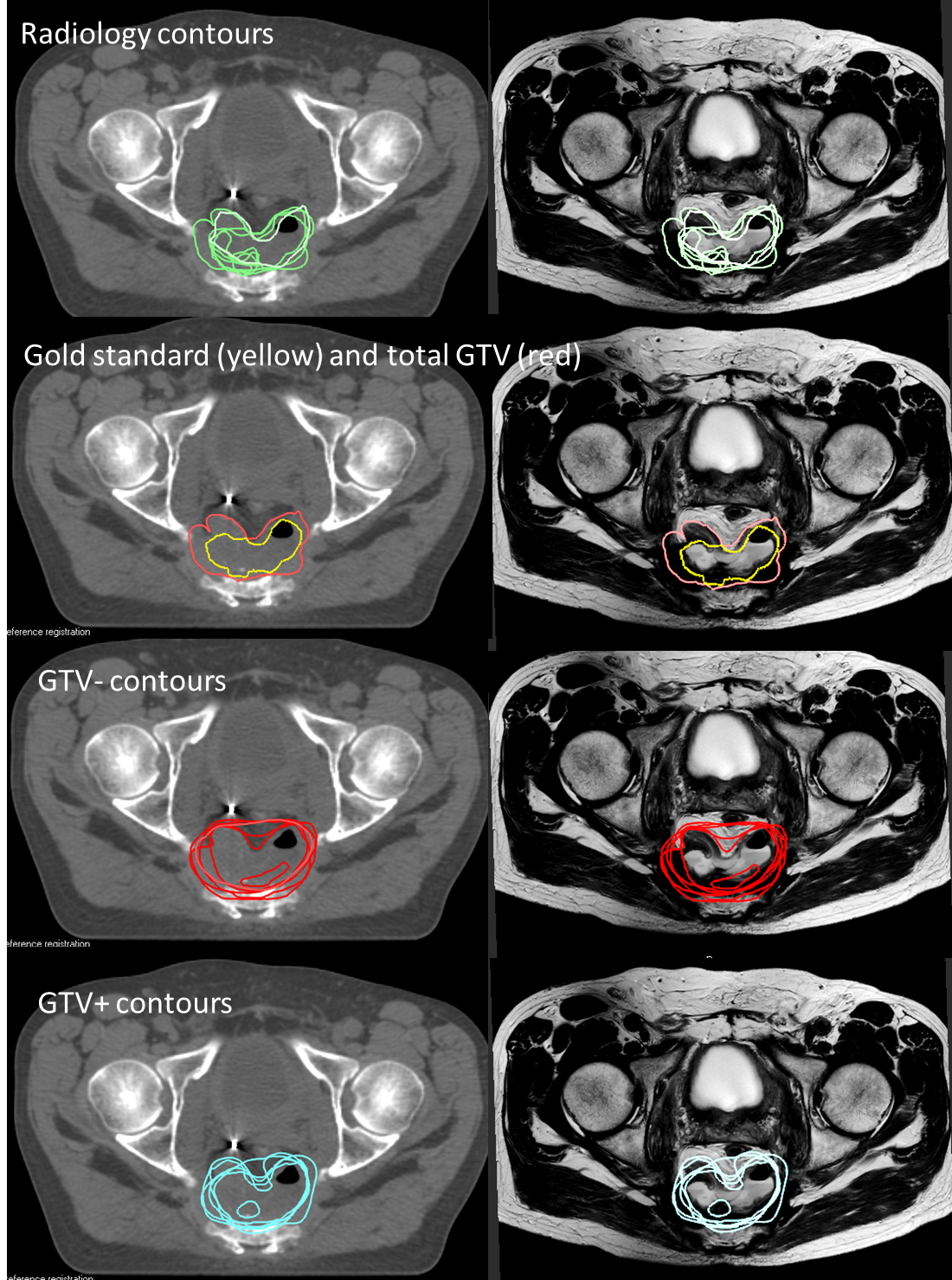


| **Case 12** | **RAD (n=8)** | | **GTV- (n=6)** | | **GTV+ (n=6)** | |
| --- | --- | --- | --- | --- | --- | --- |
|  | **Median** | **Range** | **Median** | **Range** | **Median** | **Range** |
| Volume (cc) | 53.1 | 1.4-155.6 | 180.3 | 6.3-307.1 | 115.3 | 4.0-259.6 |
| SDSC (0-1) | 0.33 | 0.08-0.79 | 0.75 | 0.01-0.91 | 0.69 | 0.00-0.98 |
| DSC (0-1) | 0.40 | 0.04-0.82 | 0.86 | 0.06-0.92 | 0.80 | 0.06-0.94 |
| HD98% (mm) | 29.2 | 17.5-58.2 | 9.2 | 4.8-69.4 | 23.4 | 3.3-52.2 |

**Case 13**

72-year old male, with a history of a T1-2N2 rectal cancer for which he received CRT, followed by a TEM due to a good response. The performed TEM was irradical, so it was followed up by an APR (ypT3N0, R0). Patient presented with a local recurrence for which he received induction chemotherapy (4 cycles of FOLFOX). He has been referred for chemo reirradiation. Patient is planned for a re-APR with IORT.

Imaging:

- *Baseline MRI:* Suspicion of a multifocal local recurrence with a solid lesion against the right seminal vesicle (22mm) and a second lesion at the perineum against the base of the penis (13mm).
- *Baseline PET-CT:* Strong suspicion of a local recurrence on the right pelvic floor. Second small focus very caudally at the anus, possibly inflammatory, but recurrence cannot be ruled out. No distant metastases.
- *MRI after induction chemotherapy:* Average response of the multifocal recurrence, with a rest lesion still visible against the seminal vesicles and sacrospinal ligament on the right and between the peritoneum and prostate on the left.
- *PET-CT after induction chemotherapy:* Little response of the recurrence on the mesorectal fascia. There is a complete response of the second lesion at the perineum left. No new lesions.

| **Case 13** | **RAD (n=8)** | | **GTV- (n=5)** | | **GTV+ (n=6)** | |
| --- | --- | --- | --- | --- | --- | --- |
|  | **Median** | **Range** | **Median** | **Range** | **Median** | **Range** |
| Volume (cc) | 2.7 | 2.0-4.0 | 8.8 | 4.3-12.6 | 5.8 | 4.0-11.1 |
| SDSC (0-1) | 0.94 | 0.75-0.99 | 0.91 | 0.80-0.93 | 0.83 | 0.67-0.99 |
| DSC (0-1) | 0.75 | 0.58-0.79 | 0.71 | 0.71-0.79 | 0.71 | 0.55-0.89 |
| HD98% (mm) | 4.9 | 3.2-39.3 | 5.6 | 4.2-15.4 | 37.4 | 3.0-40.4 |

**
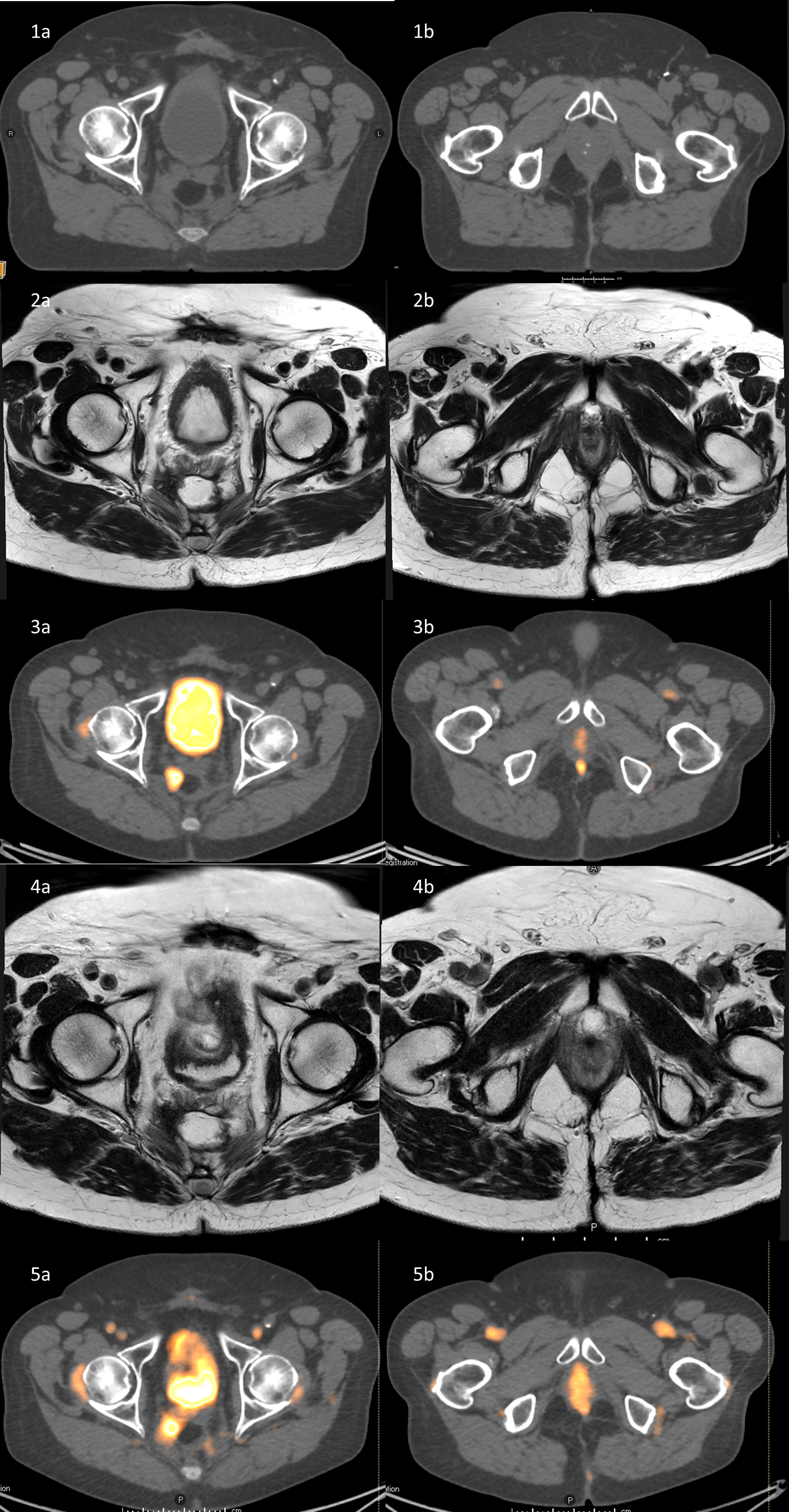
**

*Figure showing (1a+b) planning CT of (a) lesion against the mesorectal fascia and (b) at the perineum, (2a+b) baseline MRI, (3a+b) baseline PET-CT, (4a+b) MRI after induction chemotherapy (5a+b) PET-CT after induction chemotherapy.*

**
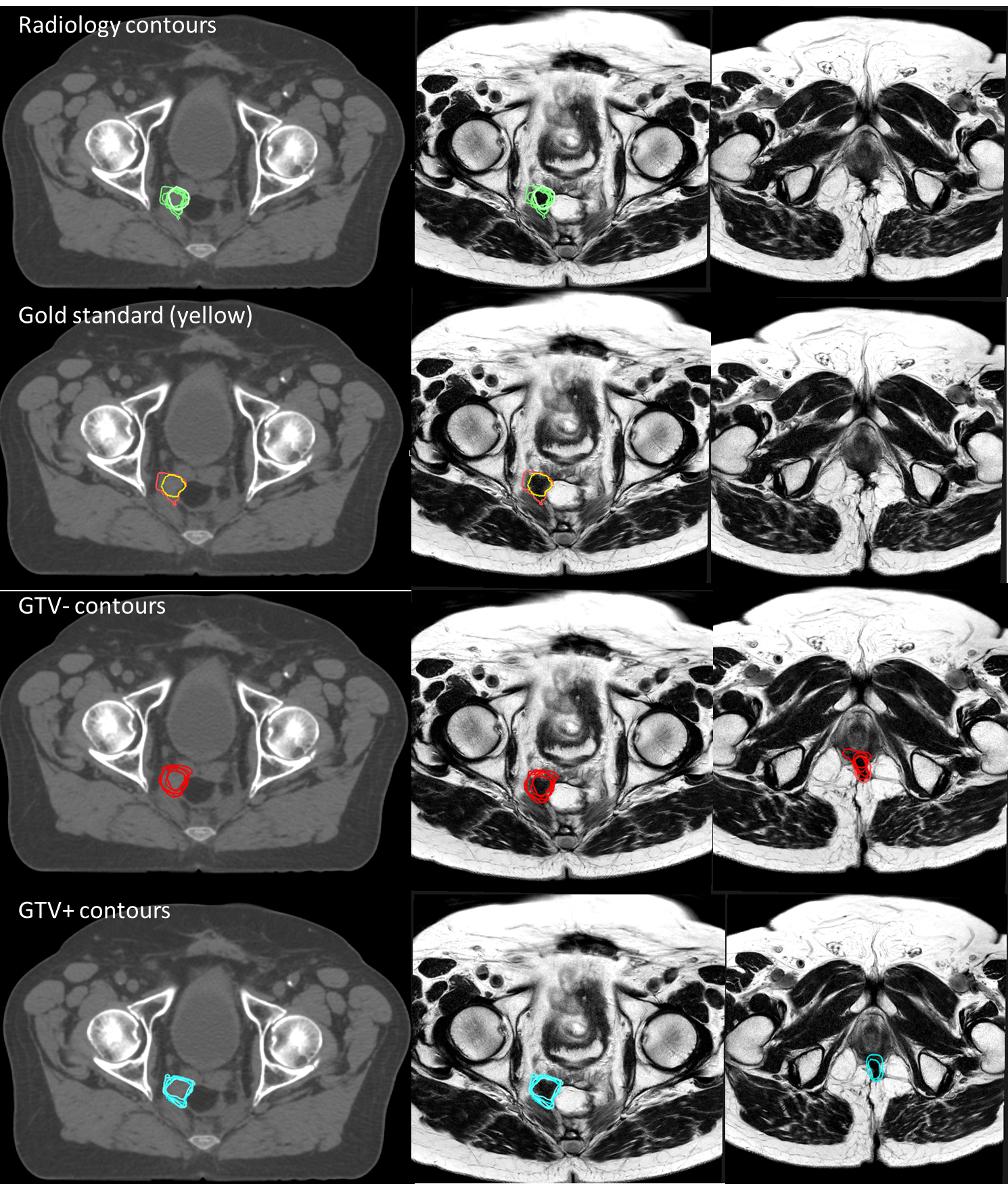
**

**Case 14**

76-year old male with a history of a T3N1 mucinous rectal cancer, for which he received 5x5Gy radiotherapy and a LAR (pT2N0, R0). Patient was diagnosed with a recurrence at the anastomosis and has been referred to you following three cycles of CAPOX induction chemotherapy. Following chemo reirradiation, patient is planned for an APR with IORT.

Imaging:

- *Colonoscopy at baseline:* At 5cm from the anorectal junction a cancerous lesion is seen (ulcerating). Biopsy: adenocarcinoma.
- *MRI at baseline:* Tumorous soft tissue visible at the anastomosis of approximately 2.6x3.2cm. There is ventral involvement of the seminal vesicles. No involvement dorsally of the mesorectal fascia.
- *PET-CT at baseline:* Known anastomotic recurrence with FDG-PET uptake circumferentially around the anastomosis for approximately 3.8cm. No suspicious local lymph nodes or distant metastases.
- *MRI after induction chemotherapy:* minimal rest lesion at the anastomosis, but still with possible involvement of the posterior side of the seminal vesicles.
- *PET-CT after induction chemotherapy:* Significant reduction of size and metabolic activity of anastomotic recurrence. Small focal metabolic activity fitting with tumorous tissue.


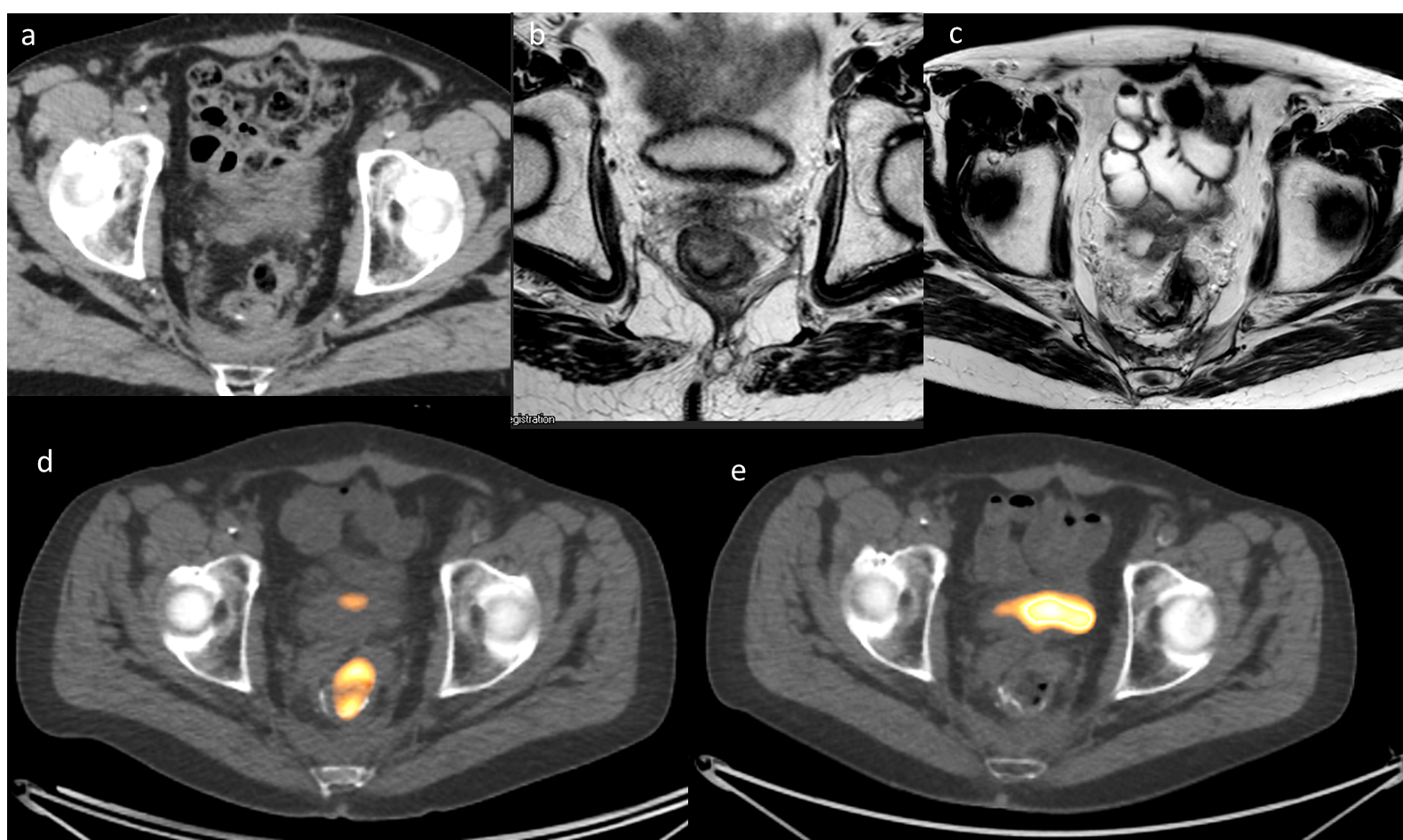


*Figure showing (a) planning CT (b) baseline MRI (c) MRI after induction chemotherapy (d) PET-CT at baseline (e) PET-CT after induction chemotherapy*


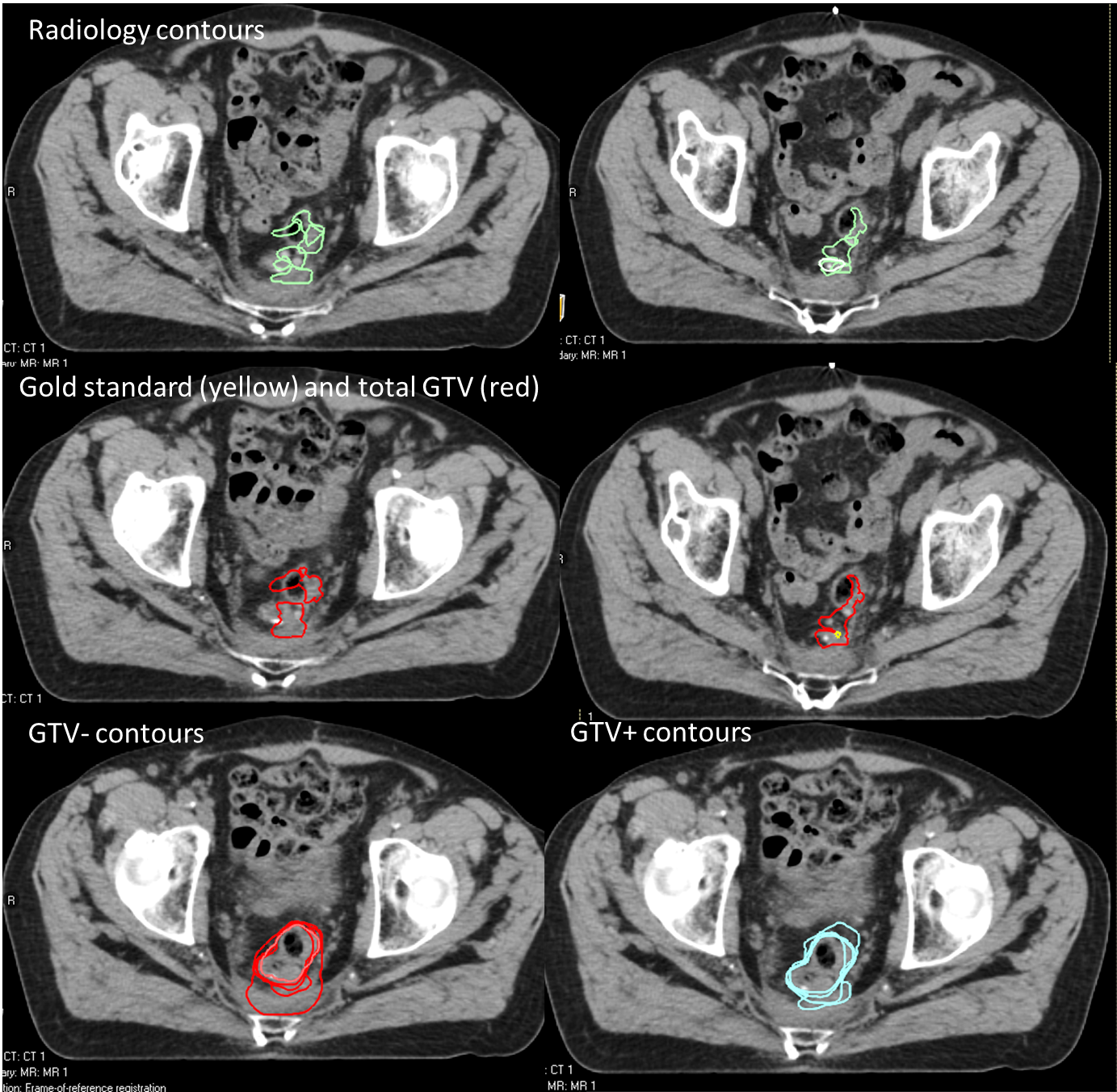


| **Case 14** | **RAD (n=8)** | | **GTV- (n=6)** | | **GTV+ (n=6)** | |
| --- | --- | --- | --- | --- | --- | --- |
|  | **Median** | **Range** | **Median** | **Range** | **Median** | **Range** |
| Volume (cc) | 2.1 | 0.9-7.8 | 34.49 | 7.4-90.7 | 32.2 | 1.7-44.3 |
| SDSC (0-1) | 0.16 | 0.00-0.60 | 0.82 | 0.26-0.91 | 0.66 | 0.10-0.94 |
| DSC (0-1) | 0.04 | 0.00-0.20 | 0.82 | 0.27-0.88 | 0.70 | 0.09-0.89 |
| HD98% (mm) | 25.6 | 8.81-45.7 | 17.5 | 5.6-26.9 | 23.3 | 6.0-26.6 |
